# Suppression of VEGFD expression by S-nitrosylation promotes the development of lung adenocarcinoma

**DOI:** 10.1186/s13046-022-02453-8

**Published:** 2022-08-08

**Authors:** Qiangqiang He, Meiyu Qu, Tingyu Shen, Yana Xu, Jiahao Luo, Dan Tan, Chengyun Xu, Muhammad Qasim Barkat, Ling-Hui Zeng, Ximei Wu

**Affiliations:** 1 Department of Pharmacology, Zhejiang University School of Medicine, 866 Yuhangtang Road, 310058 Hangzhou, China; 2 Department of Pharmacology, Zhejiang University City College, 51 Huzhou Street, 310015 Hangzhou, China; 3 Department of Orthopaedics, Sir Run Run Shaw Hospital, Zhejiang University School of Medicine, 310016 Hangzhou, China

**Keywords:** VEGFD, VEGFA, Lung adenocarcinoma, S-nitrosylation, Angiogenesis, GSNOR

## Abstract

**Background:**

Vascular endothelial growth factor D (VEGFD), a member of the VEGF family, is implicated in angiogenesis and lymphangiogenesis, and is deemed to be expressed at a low level in cancers. S-nitrosylation, a NO (nitric oxide)-mediated post-translational modification has a critical role in angiogenesis. Here, we attempt to dissect the role and underlying mechanism of S-nitrosylation-mediated VEGFD suppression in lung adenocarcinoma (LUAD).

**Methods:**

Messenger RNA and protein expression of VEGFD in LUAD were analyzed by TCGA and CPTAC database, respectively, and Assistant for Clinical Bioinformatics was performed for complex analysis. Mouse models with urethane (Ure)–induced LUAD or LUAD xenograft were established to investigate the role of S-nitrosylation in VEGFD expression and of VEGFD mutants in the oncogenesis of LUAD. Molecular, cellular, and biochemical approaches were applied to explore the underlying mechanism of S-nitrosylation-mediated VEGFD suppression. Tube formation and wound healing assays were used to examine the role of VEGFD on the angiogenesis and migration of LUAD cells, and the molecular modeling was applied to predict the protein stability of VEGFD mutant.

**Results:**

VEGFD mRNA and protein levels were decreased to a different extent in multiple primary malignancies, especially in LUAD. Low VEGFD protein expression was closely related to the oncogenesis of LUAD and resultant from excessive NO-induced VEGFD S-nitrosylation at Cys277. Moreover, inhibition of S-nitrosoglutathione reductase consistently decreased the VEGFD denitrosylation at Cys277 and consequently promoted angiogenesis of LUAD. Finally, the VEGFD^C277S^ mutant decreased the secretion of mature VEGFD by attenuating the PC7-dependent proteolysis and VEGFD^C277S^ mutant thus reversed the effect of VEGFD on angiogenesis of LUAD.

**Conclusion:**

Low-expression of VEGFD positively correlates with LUAD development. Aberrant S-nitrosylation of VEGFD negates itself to induce the tumorigenesis of LUAD, whereas normal S-nitrosylation of VEGFD is indispensable for its secretion and repression of angiogenesis of LUAD.

**Supplementary Information:**

The online version contains supplementary material available at 10.1186/s13046-022-02453-8.

## Background

Non-small cell lung carcinoma (NSCLC) is one of the leading causes of death from cancer in the world, and lung adenocarcinoma (LUAD), a prevalent subtype of NSCLC, accounts for approximate 40% of NSCLC [1]. Investigation of the pathogenesis of LUAD is thus of great importance in the prevention, diagnosis, and treatment of this disease.

Angiogenesis is a crucial step in tumor development and metastasis [2], and this process is strictly regulated by vascular endothelial growth factors (VEGFs) and their receptors (VEGFRs). VEGFs consist of VEGFA, VEGFB, VEGFC, VEGFD, VEGFE (virally encoded), and Placental growth factor (PLGF), and play diverse roles in tumorigenesis and metastasis [3]. VEGFA is one of the most important regulators of angiogenesis in a variety of cancers [4]. VEGFB and PLGF predominantly regulate hematopoiesis [5]. VEGFC and VEGFD could induce angiogenesis and lymphangiogenesis [6]. VEGFC is indispensable for lymphangiogenesis; lymphatic development is arrested in *VEGFC* knockout mice [7, 8]. However, although *VEGFD* knockout mice have no obvious phenotype [9], *VEGFD* loss-of-function reduces the lymphatic metastasis in an orthotopic in vivo model of human pancreatic cancer and attenuates the pulmonary edema in hyperoxia-induced lung injury [10, 11]. Different from the binds of VEGFA and VEGFR1 or VEGFR2 (VEGFR1 is a high-affinity tyrosine kinase VEGFA receptor, but the lower-affinity, highly homologous VEGFR2 is the main signaling receptor for VEGFA) [3, 12, 13], VEGFD and VEGFC bind to VEGFR3 (implicated in lymphangiogenesis) but can bind to VEGFR2 after proteolytic cleavage [3, 14]. VEGF-D induced VEGFR2 phosphorylation more slowly but had a more sustained effect than VEGFA [15], differential VEGFR2 activation by VEGFA and VEGFD has distinct consequences for endothelial signaling and function, activated VEGFR2 by VEGFA induces endothelial PI3K/Akt signaling and angiogenesis more than VEGFD [15, 16].

S-nitrosylation is a nitric oxide (NO)-related post-translational modification (PTM) [17]. Protein cysteine S-nitrosylation generates S-nitrosothiols through the covalent bond formation [18]. Denitrosylation, as the reverse reaction of S-nitrosylation, is the progress that reduces SNO on protein cysteine residues to sulfhydryl. Denitrosylation could be affected by various reductase systems including Thioredoxin/thioredoxin reductase (Trx/TrxR) and Nitrosoglutathione reductase (GSNOR) [19]. S-nitrosylation has a significant role in tumor angiogenesis and metastasis; S-nitrosylation of targets in tumor cells leads to metastasis via regulating epithelial to mesenchymal transition, migration, and invasion [20, 21]. Similarly, S-nitrosylation also has crucial functions in the invasion and metastasis of NSCLC [21–23], and it is a vital regulator of radiation-induced HIF-1α activation [24]. Moreover, S-nitrosylation of β-catenin promotes VEGF-induced endothelial cell permeability [25]. However, the S-nitrosylation of these proteins regulates angiogenesis via affecting VEGFA expression. We therefore raised the question of whether S-nitrosylation has a direct effect on VEGFs. In the previous research, we analyzed the possibility of S-nitrosylation of VEGFs based on GPS-SNO, and the results showed that only VEGFD in the VEGF family had the possibility of S-nitrosylation. So, we want to explore whether S-nitrosylation is involved in VEGFD regulation and its effect on LUAD.

We have a clear understanding of the VEGF family, but current research suggests that VEGFD has sufficient but insignificant effects on angiogenesis and lymphangiogenesis. There is a lack of knowledge of VEGFD in vivo. However, VEGFD, mainly expressed in the lung, is significantly suppressed in LUAD [26], this posed an interesting problem. It is of great significance to explore the reasons and mechanisms for low expression of VEGFD in angiogenesis and metastasis of LUAD. Aberrant NO, a common tumor trigger, is closely related to angiogenesis and metastasis via S-nitrosylation. S-nitrosylation of VEGFD in LUAD may solve this problem and provide new ideas for targeted tumor angiogenesis therapy.

## Materials and methods

### Bioinformatics and statistical analyses of public databases

The Cancer Genome Atlas (TCGA) data visualization web-tools, Assistant for Clinical Bioinformatics (https://www.aclbi.com/), GEPIA (http://gepia.cancerpku.cn), UALCAN (http://ualcan.path.uab.edu/), interactively analyze cancer-related genes expression and patient survival information. UALCAN provides a protein expression analysis by using data from Clinical Proteomic Tumor Analysis Consortium (CPTAC) Confirmatory/Discovery dataset. The Human Protein Atlas (https://www.proteinatlas.org/) provides tissue and cell distribution information on human proteins.

### Regents

S-Nitrosoglutathione (GSNO, N4148), DTT (D0632), and Puromycin (P8833) were from Sigma (St. Louis, MO). L-NAME HCl (S2877) and Cycloheximide (CHX, S7418) were purchased from Selleck Chemicals (Houston, TX). Cavosonstat (N91115) were from STA for Nivalis (Shanghai). TCA Protein Precipitation Kit was purchased from Sangon Biotech (Shanghai, China). Antibodies against VEGFD(C-12), FLK1 (A-1), PECAM-1 (H-3), and c-Myc were from Santa Cruz Biotechnology (Santa Cruz, CA). Antibodies against VEGF receptor 3 (VEGFR3) were from Cell Signaling Technology (Danvers, MA). Antibodies against glyceraldehyde-3-phosphate dehydrogenase (GAPDH), HA, VEGFD, and LYVE1 were from HUABIO (Hangzhou, China). The IRDye 680 and 800 secondary antibodies were from LI-COR Bioscience (Lincoln, Nebraska). Alexa555- and Alexa488-conjugated antibodies were from Molecular Probes (Eugene, OR).

### Animal models

The two most widely used animal models of lung cancer, the carcinogen urethane (Ure; ethyl carbamate)–induced and xenograft tumors, were established. Chemically induced lung cancer was provoked upon twice weekly intraperitoneal (i.p.) injections of 1 g/Kg urethane for ten consecutive weeks, as previously reported [27]. In addition to the negative control injected with saline (*n* = 5), Ure-induced lung cancer mice were performed by eight-week-old C57/BL6 female mice (Laboratory Animal Center of Zhejiang Province, Hangzhou, China), which were randomly divided into two groups (each *n* = 5). The Ure-LUAD and Ure-LUAD + N91115 mice were injected with 1 g/Kg Ure. Moreover, the Ure-LUAD + N91115 were dosed with 1.5 mg/kg N91115 weekly by tail vein injections.

The LLC cell Xenograft tumor-bearing mice stably expressing empty vector, VEGFD, and VEGFD^C277S^ mutant were established as previously described [28]. Female C57/BL6 mice at 8-week-old (each *n* = 5) were injected subcutaneously with 0.15 ml of LLC cell suspension at 1 × 10^7^ cells/ml into the left armpit. Xenografts emerged 7 days after injection, and the volumes were recorded once every two days within 24 days.

Blood samples of urethane–induced and xenograft tumors mice were collected by extracting the eyeball, which was used for Elisa assay and various blood routine tests. The lungs of Ure-induced tumor mice and xenografts were used for preparation of protein lysates, extraction of total RNA, and paraffin-embedded histological sections. Histology, immunohistochemistry, and immunostaining were performed essentially as previously reported [28–30]. All mice were housed in a specific pathogen-free (SPF) room maintained at 23 ± 2 °C with 50 ± 10% humidity. The mice have free access to tap water and regular rodent chow. All the animal experiments were approved by the Institutional Animal Care and Use Committee of Zhejiang University.

### Cell cultures

HEK293T, BEAS-2B, and LLC cells (mouse lung cancer cells) were purchased from ATCC (Manassas, VA) and cultured in DMEM medium containing 10% fetal bovine serum (FBS). Lung adenocarcinoma cell lines, including NCI-H1975, NCI-H1650, and A549 cells, were obtained from professor Yihua Wu at the department of Toxicology, Zhejiang University School of Public Health and maintained in DMEM medium containing 10% FBS [31]. All cell lines were incubated at 37℃ with 5% CO_2_. Cells were passaged for no more than 6 months and routinely assayed for mycoplasma contamination.

### Plasmids, transfection and lentiviral infection

pCDH-CMV-MCS-3Flag-copGFP-F2A-PuroR, pOTB7-PCSK5, and pCMV-SPORT6-PCSK7 were purchased from Miaolingbio (Hubei, China). Constructs expressing the interest genes were cloned by using specific primers. Plasmid with point mutation was performed by using a KOD-plus mutagenesis kit (Toyobo, Osaka, Japan) according to the manufacturer’s instruction. The mutants were verified by nucleic acid sequencing. Transient transfection was performed in cells by using Lipofectamine 2000 (Invitrogen) as described previously. The VEGFD and VEGFD^C277S^ mutant knockin lentiviral vector was constructed based on the lentiviral vector pCDH-CMV-MCS-3Flag-copGFP-F2A-PuroR. The empty vectors and lentiviral vectors were packaged in HEK293T cells by co-transfection with the packaging plasmid pPMDL and pPAX2 (4: 1: 3). The supernatant was collected and filtered with a 0.45 μm filter after 48 h. The LLC cell culture medium was replaced by a lentivirus-containing medium supplement every 6 h with 10 µg/mL polybrene. 1 µg/mL puromycin was used to screen the stable cells after 48 of lentivirus infection.

### Western blotting, co-immunoprecipitation, and biotin-switch assay

Cell lysates were prepared by using a RIPA lysis buffer (Beyotime, Shanghai, China) supplemented with Protease Inhibitor Cocktail (PIC, Sigma-Aldrich), Phenyl-methane Sulfonyl Fluoride (PMSF, Sigma-Aldrich), and Phosphatase Inhibitor Cocktail (PhoIC A and B, Sigma-Aldrich). Equal amounts of protein of each sample were analyzed using SDS-PAGE, electrophoretic transfer, immunoblotting, and chemiluminescence detection. Either 10% or 12% SDS-PAGE was used to isolate proteins with different molecular weights. The proteins were transferred to a nitrocellulose filter membrane (PALL). Antibodies and dilutions were as follows: anti-VEGFD, 1:1000; anti-PECAM-1, 1:1000; anti-LYVE1, 1:1000; anti-FLK-1, 1:1000; anti-VEGFR3, 1:1000; anti-HA, 1:1000; anti-Flag, 1:500; anti-Myc, 1:1000; anti-GAPDH, 1:5000; anti-GSNOR, 1:2000; The Odyssey Infrared Imaging System (LI-COR Bioscience, Lincoln, Nebraska) was used to detect the immunoreactive signals. Western blot bands were quantified by measuring integrated density using the Image-J software (NIH, USA). Co-immunoprecipitation was performed through the cell lysates, and the supernatants were incubated with antibody and protein A/G PLUS-Agarose (Santa Cruz) overnight at 4℃. The bound proteins were eluted in the loading buffer and subjected to the SDS-PAGE. S-nitrosylated proteins were detected by Pierce™ S-nitrosylation Western Blot Kit (Thermo).

### Immunofluorescence staining

After transfection or lentiviral infection, cells were fixed with 4% paraformaldehyde and permeabilized with 0.1% Triton X-100. Then, Cells were washed with PBS three times and blocked with 1% BSA for 1 h. Primary antibody incubation or negative control was carried out at 4 ◦C overnight. After incubation, cells were washed three times with TBST and then incubated with either Alex Fluor 488- or 546-labeled secondary antibody (1:1000) for 1 h. Nuclei were counterstained with 4′,6-diamidino-2-phenylindole (DAPI), and images were captured by an Olympus FV3000 confocal microscope and analyzed through the FV31S-SW Viewer.

### RNA isolation and quantitative RT-PCR

Total RNA from cells or tissues was extracted using TRIzol reagent (Takara Biotechnology, Dalian, China) and reverse transcription was performed using the PrimerScript RT Reagent kit (Vazyme Biotech, Nanjing, China) as described previously [32]. Quantitative RT-PCR was performed with the BioRad CFX96 system. The primers used for the qPCR as follows: *Vegfa* forward: GATCAAACCTCACCAAAGCC, reverse: TCTTTCTTTGGTCTGCATTCAC; *Pecam1* forward: ACATAACAGAGCTGTTTCCCA, reverse: AGGACAGGTCCAACAACTC; *Prox1* forward: ACGTGAAGTTCAACAGATGC, reverse: CGCATACTTCTCCATCTGGA; *Mmp7* forward: CCACTGAACTTCAAGAGGG, reverse: GAAGCTGTCTCCATGATCTC; *Tie1* forward: CAAAGGTGACACTGCTGTG, reverse: AGTAGGATCCGTTGTTCTTCC. *PROX1* forward: AATGACTTTGAGGTTCCAGAG, reverse: TCTTTGCCTGCGATAATGG; *MMP7* forward: GCCAGATGTTGCAGAATACTC, reverse: TATGATACGATCCTGTAGGTGAC; *TIE1* forward: TGAGAAGCAGTTCATCCAC, reverse: AAGTCTGCAATCTTGGAGG; *FURIN* forward: AACACCTGGTGGTACAGAC, reverse: TATGAGTGGCTCACTTTCCG; *PC5* forward: GAAGCCAATCCGTTTCTGAC, reverse: ATTAGCGTTCAAATGTCCCG; *PC7* forward: ATGAGTCATTCCAGGTCGG, reverse: CTCTAACAGCCTTTGTCTGTC; *KLK3* forward: GTATTTCAGGTCAGCCACAG, reverse: GGTTCAATGTGGAGTCATCAC; *CTSD* forward: TCACAGTCGTCTTCGACAC, reverse: TACTTGTGGTGGATCCAGC. The mRNAs were normalized to GAPDH and β-actin, and the differences in mRNA levels were calculated by the 2^− ΔΔCt^ method.

### ELISA assay

The culture fluids were harvested and cleared of cell debris. Analyses of secreted proteins outside cells were performed using the enzyme-linked immunosorbent assay (ELISA) of VEGFD by its kits (MULTI SCIENCES, Hangzhou, China) according to the manufacturer’s instructions. Serums were collected by extracting the eyeball and subsequently centrifuging. Mouse serum VEGFD was measured with a mouse serum VEGFD ELISA kit (FANKEW, Shanghai, China). VEGFD levels of culture fluids and serum were normalized to the protein levels.

### Structural modeling

To investigate the structural and functional changes caused by mutation, a three-dimensional computer model was used for analyzing the structural location of Cys277 in VEGFD. The structure of VEGFD (273-315aa) was modeled by Modeller (https://salilab.org/modeller/registration.html) and then analyzed by PyMOL 2.2 (https://pymol.org/2/) as described previously [33, 34]. AlphaFold (https://alphafold.ebi.ac.uk/) further predicted the possible structure of VEGFD.

### Tube formation, wound healing assay, and Transwell co-culture

HUVECs from ATCC (Manassas, VA) were prepared and cultured in DMEM media (Gibco, Frederick, MD) containing 10% fetal bovine serum (FBS). After lentiviral infection, tube formation was performed by Matrigel (Solarbio, Beijing, China)-coated 24-well plate as reported [35, 36]. The tube structures were observed under a Nikon-TS100 (Nikon, Tokyo, Japan) microscope after 12 h, taking the picture. The branch length and points were counted by Image J software. Images were analyzed using Angiogenesis Analyzer, a plugin developed for the ImageJ software.

Co-culture of HUVEC and NCI-H1975 cells was performed as co-culture in Corning Inc. transwell chamber. A wound healing assay was performed to examine the effects of secreted proteins from NCI-H1975 cells on HUVEC cell migration. The NCI-H1975 cells transfected with Vector, WT, and C277S mutant were seeded in the upper chamber of 0.4 μm, and the HUVEC cells suspension containing 4 × 10^4^/mL cells were seeded on a 24-well plate, cells were co-cultured for 24 h. A wound was performed through a sterile 200µL pipette tip when the cells in each well reached 90–100% confluence. Cells were washed with PBS until there were no floating cells on the scratch and were cultured with medium without FBS for 24 h. Images were captured by Nikon-TS100 microscope. Finally, the average width of each scratch was analyzed by the Image J software. In transwell migration assay, the HUVEC cells suspension containing 2 × 10^4^/mL cells were seeded in the upper chamber of 8 μm, the NCI-H1975 cells transfected with Vector, WT, and C277S mutant were seeded on a 24-well plate; After incubation for 24 h, cells in the upper compartment were removed, the upper chamber was fixed and stained with 1% crystal violet for 20 min. Images were captured by Nikon-TS100 microscope.

### Statistics

Each experiment was repeated at least three times, and a representative result is shown. Numerical data were presented as means ± SD. Statistical analyses were performed by the SPSS statistical package (IBM, North Castle, NY). Statistical significance was assessed at levels of *p* < 0.05 and p < 0.01 by one-way ANOVA and the Tukey-Kramer multiple comparison test or by Student’s t-test.

## Results

### VEGFD is inhibited in LUAD and is correlated with LUAD development

**Fig. 1:**
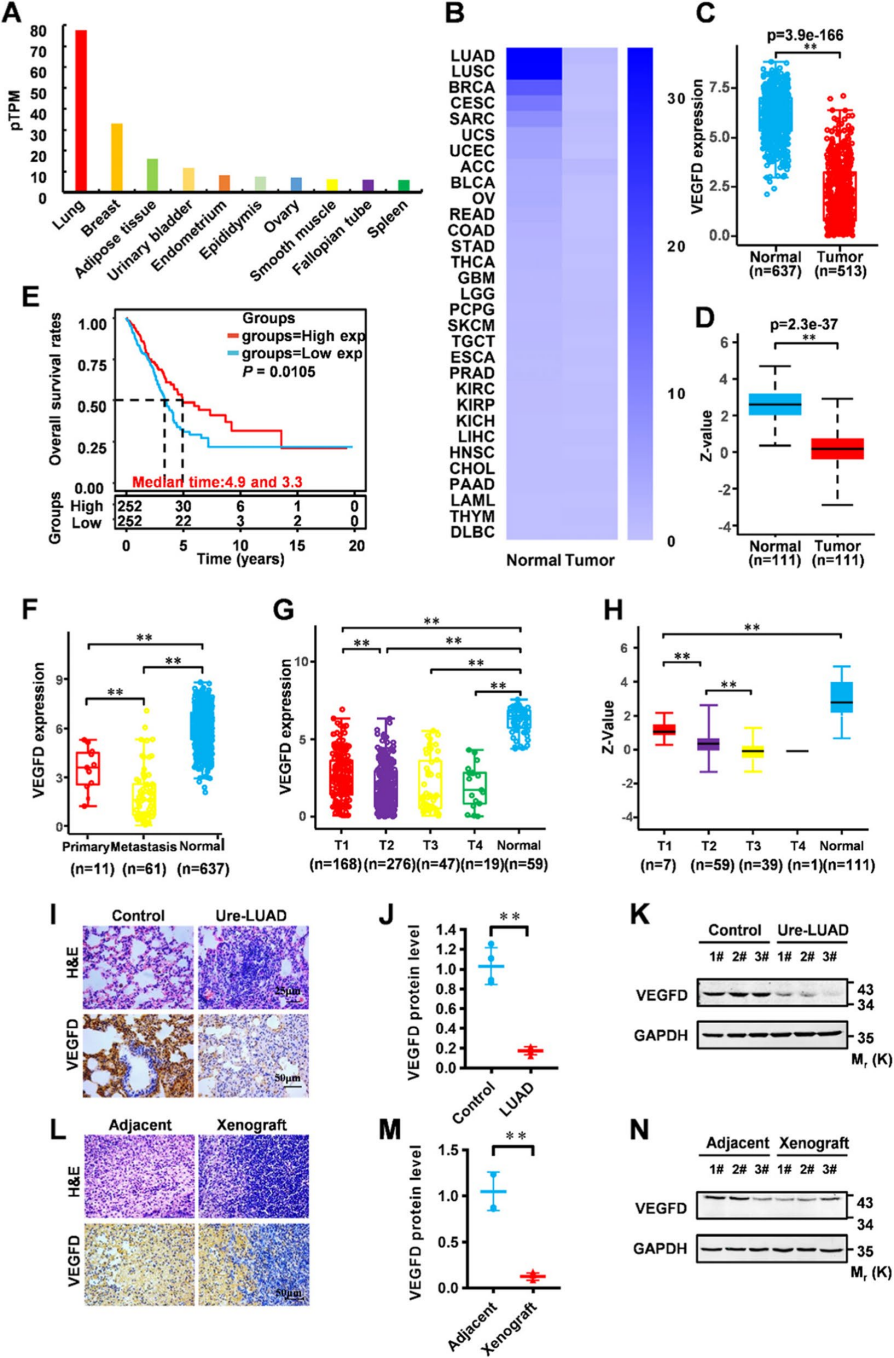
The expression patterns and clinical significance of VEGFD in lung adenocarcinoma. **A** *VEGFD* mRNA expression patterns extracted from the database of The Human Protein Atlas. **B** *VEGFD* mRNA expression patterns in tumors extracted from the database of TCGA. **C** *VEGFD* mRNA expression patterns in lung adenocarcinoma extracted from the database of TCGA. **D** VEGFD protein expression patterns in lung adenocarcinoma extracted from the database of CPTAC. **E** Kaplan‑Meier survival curves of patients with lung adenocarcinoma expressing different levels of *VEGFD* gene from the database of Assistant for Clinical Bioinformatics. **F**, **G** *VEGFD* mRNA expression patterns in different stages and sites of tumor development extracted from the database of Assistant for Clinical Bioinformatics. **H** VEGFD protein level in different stages and sites of tumor development extracted from the database of CPTAC. **I**-**J** Lungs of urethane-induced lung tumors were used for sectioning, H&E staining, IHC for VEGFD, and their quantification. Numerical data were expressed as mean ± SD (each *n* = 3). **K** Western blot analyses for VEGFD in urethane-induced lung tumors. **L-M** Xenografts were used for sectioning, H&E staining, IHC for VEGFD, and their quantification. Numerical data were expressed as mean ± SD (each *n* = 3). **N** Western blot analyses for VEGFD in LLC cell xenografts. Data in (**K**) and (**N**) are representative of three independent experiments, and data in (**J**) and (**M**) represent the mean ± SEM of triplicate samples. *P < 0.05, **P < 0.01, Student’s t test

First, we analyzed the expression of VEGFD to interrogate the role of VEGFD in the specific tumor. We found that VEGFD was specifically and highly expressed in the lungs through the human protein atlas database (Fig. 1A). The TCGA analysis showed that *VEGFD* mRNA was decreased in multiple cancers, especially lung adenocarcinoma (LUAD) (Fig. 1B and C). To verify the protein level of VEGFD in LUAD, CPTAC analysis showed a reduced protein level of VEGFD in LUAD (Fig. 1D). Next, we analyzed the effect of VEGFD mRNA level on LUAD patient survival through Assistant for Clinical Bioinformatics. The consequences showed that the median survival time of LUAD patients with high VEGFD mRNA and patients with low VEGFD was 4.9 years and 3.3 years, and the survival probability of LUAD patients with high expressed VEGFD was significantly higher than patients with low VEGFD (Fig. 1E). To clarify the correlation between VEGFD and LUAD development, a comparison of the VEGFD mRNA in patients with primary and metastatic LUAD and normal human VEGFD showed that VEGFD mRNA level significantly decreased once tumor metastasis (Fig. 1F). Similarly, we inspected the VEGFD mRNA level in different tumor stages of LUAD (T1-T4). The Assistant for Clinical Bioinformatics showed that the VEGFD mRNA level did not decline with the tumor development (Fig. 1G). Interestingly, CPTAC analysis showed that VEGFD protein level decreased significantly with the LUAD development (Fig. 1H). Those results indicated that the VEGFD protein level was significantly reduced in LUAD and was closely related to tumor development.

To prove these bioinformatics results, we constructed a lung cancer mice model induced by urethane. The H&E and immunohistochemistry (IHC) results of the lung tissue section showed that VEGFD protein was significantly low expressed in the lung cancer mice model (Fig. 1I J). Western Blot analysis showed that the protein abundance of VEGFD was remarkably decreased in LUAD mice compared with normal (Fig. 1K). We also constructed a murine subcutaneous transplantation model of mouse lung cancer cells (LLC). The H&E and IHC results of tumor tissue sections also showed that the protein abundance of VEGFD in tumor tissues was significantly lower than that in adjacent tissues (Fig. 1L and M). Western blot analysis also showed that the protein level of VEGFD was unusually decreased in tumor tissues compared with adjacent (Fig. 1N).

Based on the above bioinformatics and mouse model results, we inferred that VEGFD protein level is impressively repressed in LUAD. Furthermore, the low-expressed VEGFD protein level is closely related to the tumorigenesis and metastasis of LUAD.

### Excessive NO in LUAD inhibits VEGFD

**Fig. 2:**
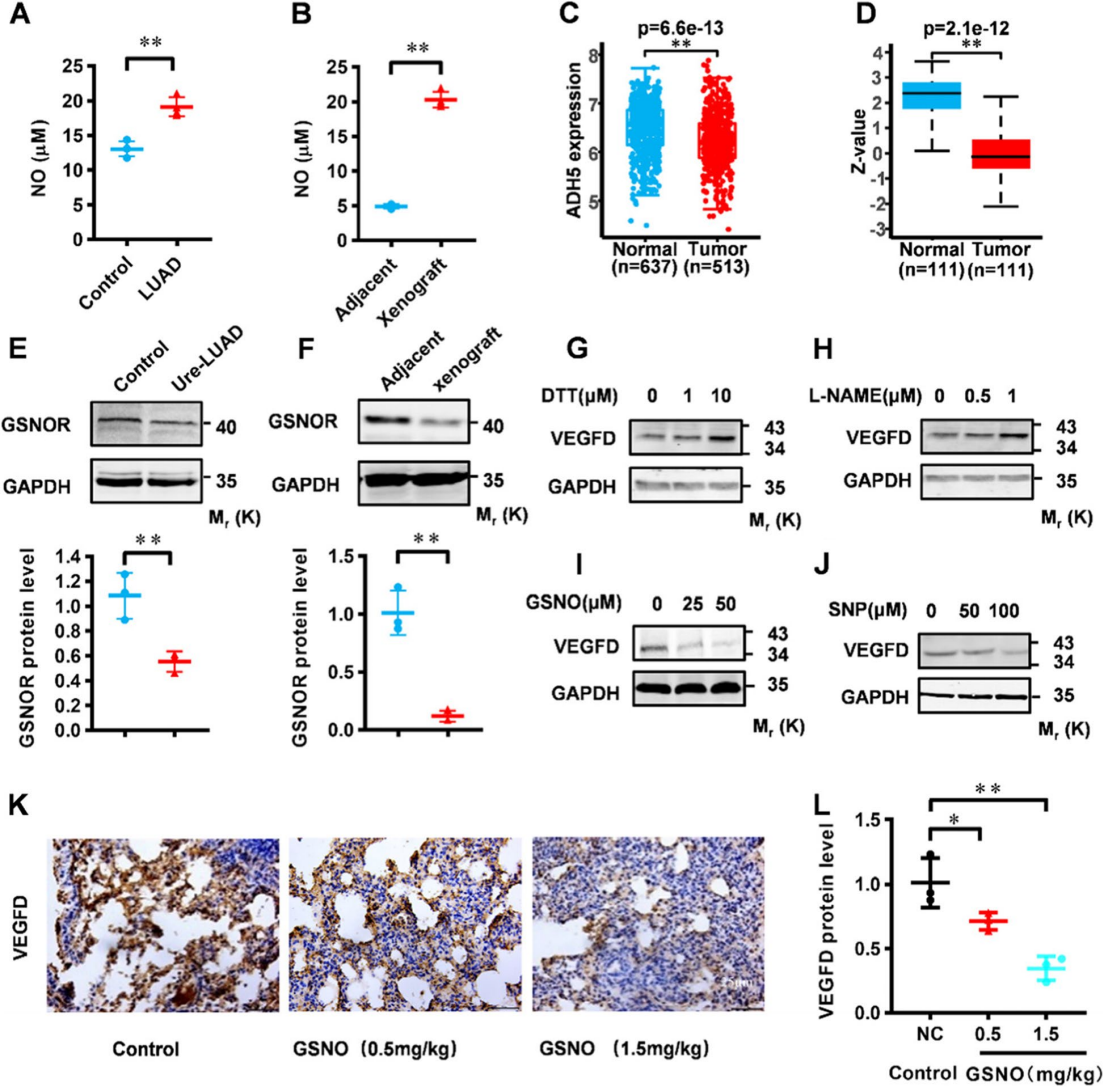
NO suppresses VEGFD in protein level. **A** NO levels in lungs with urethane-induced tumors, quantification was based on 3 samples of each group. **B** NO levels in LLC cell xenografts, quantification was based on 3 samples of each group. **C** The mRNA level of *ADH5* in lung adenocarcinoma extracted from the database of TCGA. **D** The Protein level of GSNOR in lung adenocarcinoma extracted from the database of CPTAC. **E-F** Western blot analysis of VEGFD protein levels in carcinogen urethane–induced and LLC xenograft tumors, and their quantification. Numerical data were expressed as mean ± SD (each *n* = 3). **G-H** Western blot analyses in NCI-H1975 cells treated with DTT, L-NAME, GSNO or SNP at the indicated concentrations for 24 h. **K-L** GSNO was administered to eight-week-old C57/BL6 female mice at the doses of 0.5 and 1.5 mg/kg (blank control) by single intratracheal instillation, lungs were used for sectioning. IHC for VEGFD, and their quantification. Numerical data were expressed as mean ± SD (each *n* = 3). Data in (**G**), (**H**), (**I**), and (**J**) are representative of three independent experiments, and data in (**A**), (**B**), (**E**), and (**F**) represent the mean ± SEM of triplicate samples. *P < 0.05, **P < 0.01, Student’s t test

After clarifying the correlation between VEGFD and LUAD, we try to explore the mechanism of inhibition of VEGFD protein level. Among numerous triggers of lung cancer, the level of NO in the tumor microenvironment is closely related to the metastasis of lung cancer [20, 22]. To examine the relationships between NO and VEGFD, we firstly measured NO concentration in lung tissue through a NO assay kit. The results showed that the NO concentration of the Urethane (Ure)-LUAD model significantly increased in the lung tissue compared with normal mice (Fig. 2A). The determination of NO concentration in the LLC xenograft tumor model also showed that NO in tumor tissues was significantly higher than in adjacent tissues (Fig. 2B). GSNOR partakes in denitrosylation in multiple organisms [37], *Gsnor* knockout mice have significantly increased nitroso levels [38]. The TCGA and CPTAC analysis results showed that the mRNA and protein level of GSNOR in LUAD was significantly decreased (Fig. 2C and D). Western blot results verified the suppression of GSNOR in the Ure-LUAD (Fig. 2E), which was similar to the xenograft model (Fig. 2F). These results indicated that NO was increased appreciably in LUAD.

To confirm the function of excessive NO on VEGFD. In vitro, NCI-H1975 cells, a LUAD cell line, were treated with DTT (inhibitor of NO/S-nitrosylation) at different doses. Western blot analysis showed that DTT can activate VEGFD at protein level (Figs. 2G and S1A). Similarly, we treated NCI-H1975 cells with L-NAME to inhibit NO. Western blot analysis also showed that inhibition of NO can activate VEGFD (Fig. 2 H and S1B). Instead, we treated the cells with GSNO, and the result of Western blot showed that GSNO could significantly inhibit VEGFD at protein level (Figs. 2I and S1C). Likely, SNP, another NO donor, could repress the protein level of VEGFD (Figs. 2 J and S1D). The above results suggested that NO repressed VEGFD protein in vitro. Moreover, the NO level is closely related to cell density. The abnormal proliferation of tumor cells induced NO production by cell metabolism at high cell density (Fig. S1E). Western blot results showed that the expression of GSNOR and VEGFD at high cell density also decreased significantly (Fig S1F). Those suggested that the increase in NO was accompanied by the inhibition of VEGFD at high cell density.

To verify the above conclusions in vivo, GSNO was administered to eight-week-old C57/BL6 female mice at the doses of 0.5 and 1.5 mg/kg (blank control) by single intratracheal instillation, IHC staining of VEGFD indicated that GSNO significantly suppressed VEGFD protein in the lung (Fig. 2K and L). Further, we constructed an acute lung injury (ALI) model induced by intratracheal instillation of Lipopolysaccharide (LPS), which is believed to be accompanied by an increase in NO concentration [39]. The NO assay kit detected an increased NO level in the ALI model compared to the control (Fig. S1G). The H&E staining of lung tissue sections showed that LPS induced lung injury (Fig. S1H). The IHC results indicated that the protein level of VEGFD in the LPS group was significantly lower than that in the control group, while expression of VEGFD in the LPS + GSNO group decreased compared with the LPS group (Fig. S1I), suggesting that GSNO inhibits VEGFD in vivo. Therefore, excessive NO inhibits VEGFD protein levels in vivo and in vitro.

### NO represses VEGFD via S-nitrosylation

**Fig. 3:**
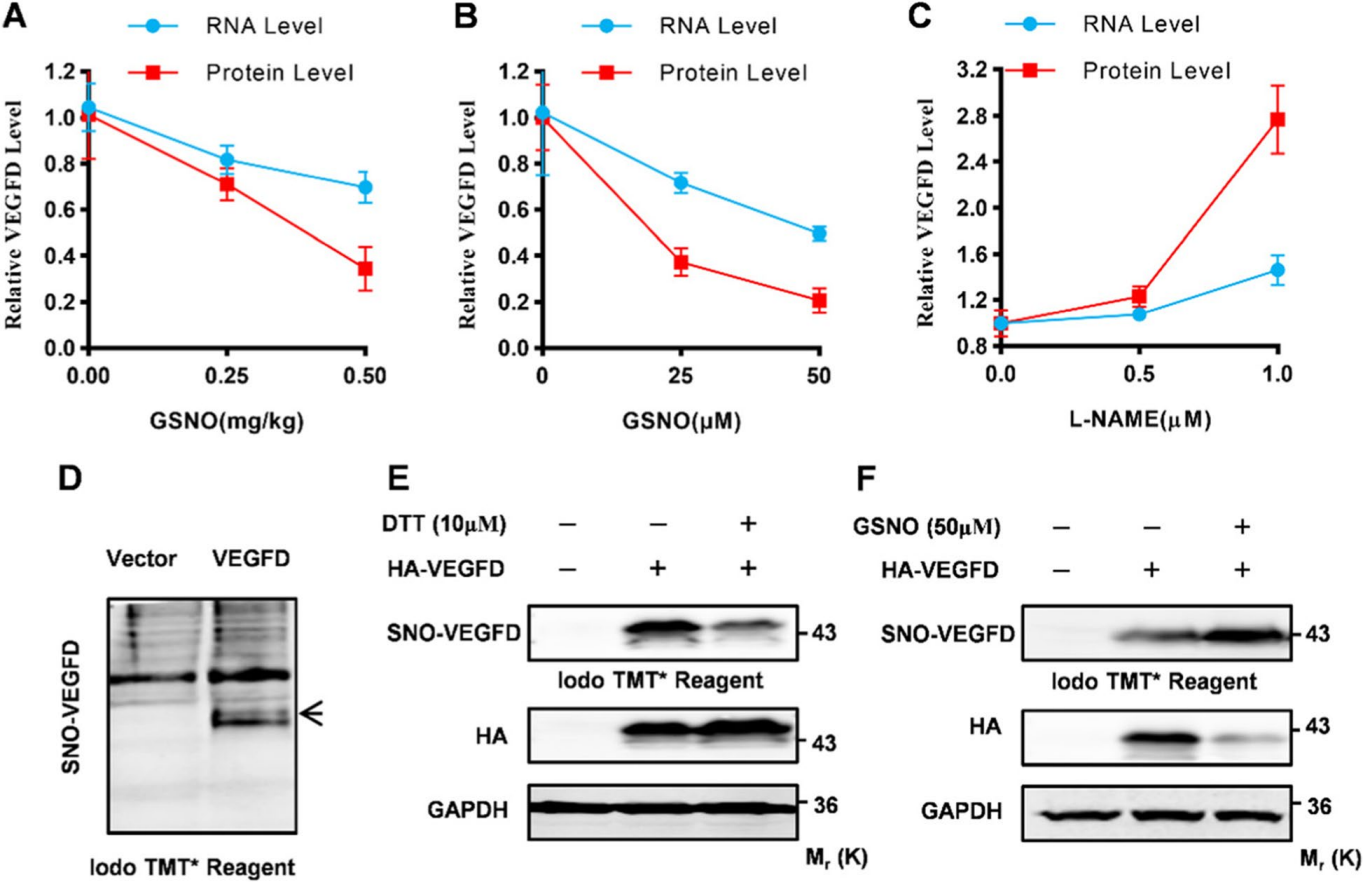
VEGFD is S-nitrosylated. **A** GSNO was administered to eight-week-old C57/BL6 female mice at the doses of 0.5 and 1.5 mg/kg (blank control) by single intratracheal instillation, RNA and Proteins were isolated from lungs. mRNA level and quantification of VEGFD protein level using Western Blot analysis (*n* = 3 per group). **B-C** NCI-H1975 cells treated with GSNO or L-NAME. qRT-PCR, quantification of WB analysis for VEGFD. Numerical data were expressed as mean ± SD (each *n* = 3). **D** HEK-293T cells were transfected with HA-VEGFD, cell extract was harvested 48 h after transient transfection. Thermo Scientific™ Pierce™ S-Nitrosylation Western Blot Kit detects the S-nitrosylated VEGFD. **E-F** HEK-293T cells transfected with vector or VEGFD-expressing construct, cultured for 24 h, and then treated with DTT (10µM) or GSNO (50µM) for further 24 h, Thermo Scientific™ Pierce™ S-Nitrosylation Western Blot Kit detects the S-nitrosylated VEGFD in cell lysate. Data in (**D**), (**E**), and (**F**) are representative of three independent experiments, and data in (**A**), (**B**), and (**C**) represent the mean ± SEM of triplicate samples. *P < 0.05, **P < 0.01, Student’s t test

For the mechanism of NO acts on the organism, as we have previously reported [18], there are mainly two ways: On one side, NO acts as a signal to affect downstream signal transduction; on the other side, NO influences proteins involved in tumorigenesis through S-nitrosylation. We found that protein levels of VEGFD in LUAD decreased more significantly than mRNA, suggesting the existence of protein post-translational modification. To verify the action of VEGFD post-translational modification, we compared the mRNA and protein levels of VEGFD in the lungs of C57/BL6 female mice administered GSNO, the result indicated that NO may suppress VEGFD via S-nitrosylation (Fig. 3A). Furthermore, NCI-H1975 cells were treated with GSNO or L-NAME at different doses, and the changes in mRNA and protein levels of VEGFD at the same time and dose were less consistent (Fig. 3B, C). The inconsistent results indicated that VEGFD may undergo S-nitrosylation.

Moreover, HEK-293T cells were transfected with thioredoxins and GSNOR, two main denitrosylation enzymes, the Western blot results show denitrosylation could activate VEGFD protein (Fig. S2A). Subsequently, the Acyl-biotinyl Exchange (ABE) assay through Thermo Scientific™ Pierce™ S-nitrosylation Western Blot Kit detects the S-nitrosylated VEGFD (Fig. 3D). The ABE assay showed that GSNO promoted the S-nitrosylation of VEGFD while DTT inhibits the S-nitrosylation (Fig. 3E F). These results suggested that VEGFD is S-nitrosylated. Meanwhile, we also explore the functions of S-nitrosylation on VEGFA, VEGFB, and VEGFC. Similarly, simultaneous transfection of HEK-293T cells with other VEGFs members (VEGFA, VEGFB, and VEGFC) and GSNOR and Trx, the Western blot results showed that denitrosylation has no effects on the expression of VEGFA, VEGFB, and VEGFC (Fig. S2B, C, and D). These further indicated that NO have a specific role in VEGFD via S-nitrosylation, differ from other VEGFs.

### Cysteine 277 in VEGFD is S-nitrosylated

**Fig. 4:**
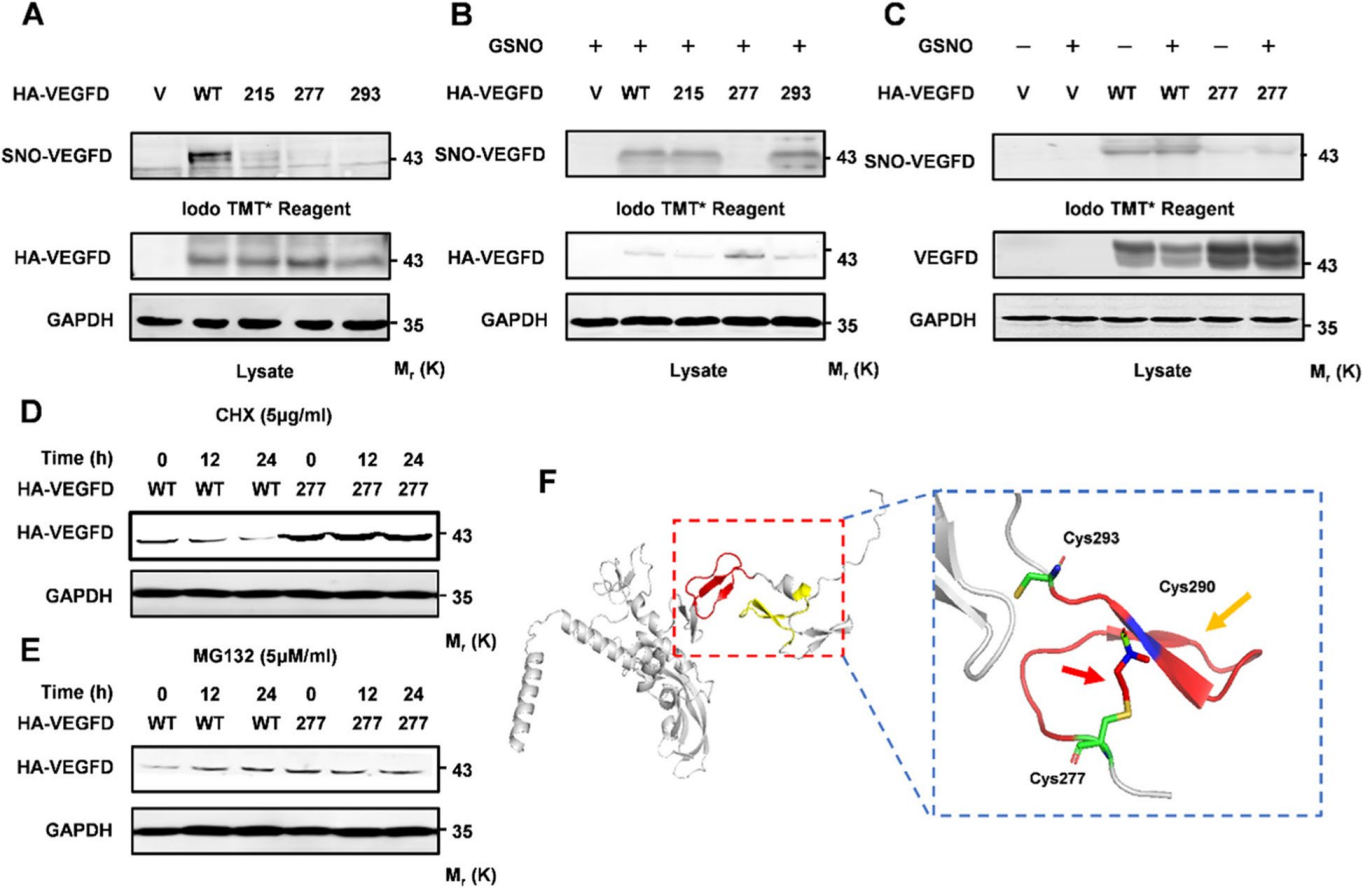
VEGFD is S-nitrosylated at Cys277. **A-C** HEK-293T cells were transfected with wild type VEGFD (WT) or its S-nitrosylation-deficient mutants, cultured for 24 h, and then treated with or without 50 µM GSNO for further 24 h, Acyl-biotinyl exchange (ABE) and Western blotting analysis VEGFD. **D-E** HEK-293T cells were transfected with WT or VEGFD(C277S) mutant, cultured for 24 h, and then treated with 5 µg/µL CHX or 5µM MG132 at different times for the indicated times, VEGFD were detected by western blot analysis. **F** Structural modeling of VEGFD (residues 273–315) and the molecular contacts around Cys277. Data in (**A**), (**B**), (**C**), (**D**), and (**E**) are representative of three independent experiments

We have confirmed by in vitro and in vivo experiments that NO inhibits VEGFD through S-nitrosylation. The S-nitrosylation of proteins occurs at the cysteine residues, Cys215, Cys277, and Cys293 may be the potential S-nitrosylation sites through GPS-SNO prediction. We created a series of VEGFD Cys to Ser mutants (HA-VEGFD^C215S^, HA-VEGFD^C277S^, and HA-VEGFD^C293S^), and their S-nitrosylation levels were characterized by ABE assays. All of these HA-VEGFD mutants decreased S-nitrosylation levels (Fig. 4A). However, only the VEGFD^C277S^ mutant affected the expression of VEGFD (Fig. 4A). To verify that S-nitrosylation suppresses VEGFD at Cys277, all HA-VEGFD mutants were stimulated with GSNO. The ABE assay indicated that the VEGFD^C277S^ mutant couldn’t be S-nitrosylated by GSNO (Fig. 4B C). Western blot analysis showed that only the VEGFD^C277S^ mutant has increased (Fig. 4B). The suppression of VEGFD by S-nitrosylation was relieved in the VEGFD^C277S^ mutant (Fig. 4C). These results indicated that VEGFD is S-nitrosylated at Cys277. However, the mechanism of S-nitrosylation of VEGFD at Cys277 is unknown. NCI-H1975 cells, transfected with HA-VEGFD and VEGFD^C277S^ mutant, were treated with cycloheximide (CHX). Western blot results showed that the VEGFD^C277S^ mutant degradation was significantly reduced (Fig. 4D). Similarly, NCI-H1975 cells, transfected with HA-VEGFD and VEGFD^C277S^ mutant, were treated with MG-132. Western blot results showed that the VEGFD^C277S^ mutant stability was significantly reduced (Fig. 4E). Thence, we speculate that S-nitrosylation decreases the stability of VEGFD. To better understand the potential influence of S-nitrosylation on the protein structure of VEGFD, we performed structural modeling. Although the crystal structure of VEGFD has not yet been identified, the crystal structure (PDB ID:1KMX) of VEGFA has been well established. Sequence alignment of VEGFD by Swiss Model indicated that VEGFD has a similar heparin-binding domain as VEGFA, which allows us to use VEGFA as a template to model the carboxyl-terminal region of VEGFD. We then modeled the spatial structure of the carboxyl-terminal (residues 275–315) of VEGFD. Structural modeling analyses showed that a disulfide bond formed between Cys277 and Cys290 (Fig. 4F). To further confirm our prediction, our predicted structure of VEGFD is consistent with the predicted structure through AlphaFold [40, 41]. In a word, S-nitrosylation inhibits VEGFD by influencing its stability.

### GSNOR restricts the S-nitrosylation of VEGFD

**Fig. 5:**
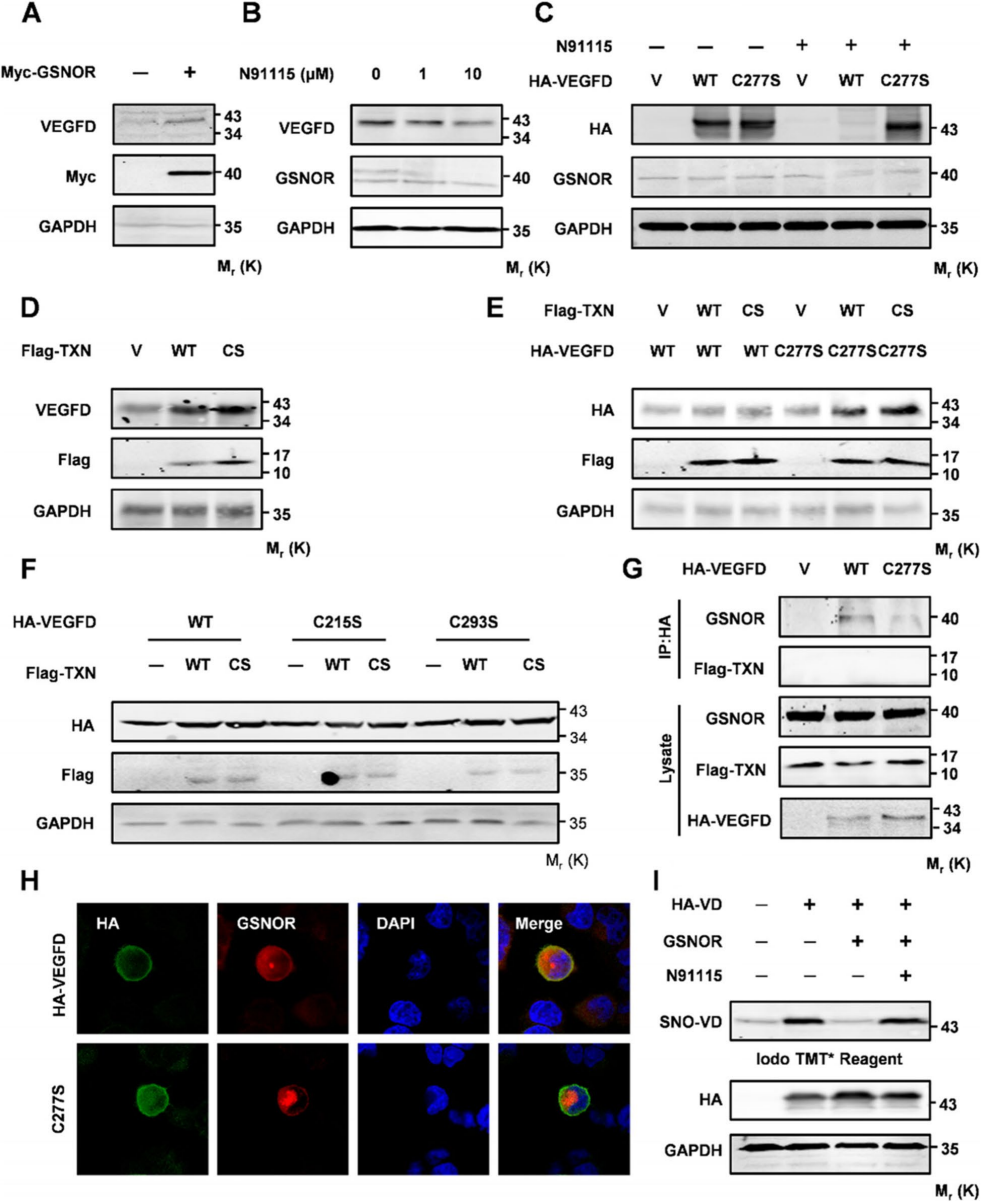
GSNOR but not Trx-1 is responsible for denitrosylation of VEGFD. **A** NCI-H1975 cells were transfected with Vector or Myc-GSNOR, cells were collected 48 h later, Western Blot was used to analyze VEGFD. **B** Western blot analyses in NCI-H1975 cells treated with the increasing concentrations of N91115 for 24 h. **C** NCI-H1975 cells were transfected with HA-VEGFD(WT) or HA-VEGFD(C277S), cultured for 24 h, and then treated with N91115 at 10 µM for further 24 h, Western Blot was used to analyze VEGFD. **D** Western blot analyses in NCI-H1975 cells transfected with vector, Flag-Trx-1(WT) or its inactive mutant Flag-Trx-1(CS) and then cultured for 24 h. **E-F** NCI-H1975 cells were transfected with HA-VEGFD(WT)/HA-VEGFD(C277S)/ HA-VEGFD(C215S)/ HA-VEGFD(C293S) in combination with Flag-Trx-1(WT)/Flag-Trx-1(CS), cell extract was harvested *48 h* after transient transfection, Western Blot was used to analyze VEGFD. **G**-**H** Co-immunoprecipitation analyses and immunofluorescence staining in NCI-H1975 cells at 24 h post-transfection with HA-VEGFD(WT) and HA-VEGFD(C277S). **H** ABE and western blot analyses for detection of S-nitrosylated VEGFD and VEGFD in NCI-H1975 cells co-transfected with HA-VEGFD and GSNOR, cultured for 24 h, and then treated with N91115 at 10 µM for further 24 h. Data in (**A**), (**B**), (**C**), (**D**), (**E**), (**F**), (**G**), and (**I**) are representative of three independent experiments

The S-nitrosylation of protein is a reversible redox process. The process of reducing SNO on protein cysteine residues to sulfhydryl is called denitrosylation. Nitrosoglutathione reductase (GSNOR) and thioredoxin/thioredoxin reductase (Trx/TrxR) are two significant enzyme systems. To investigate the mechanism of denitrosylation in VEGFD, we first explore the function of GSNOR in VEGFD. Overexpression of GSNOR increased the VEGFD in protein level (Fig. 5A). Instead, A549 cells were treated with N91115 (a kind of GSNOR inhibitor). Western blot results suggested that time- and dose-dependent effects of N91115 repressed VEGFD (Fig. 5B C). Meanwhile, to verify whether GSNOR is involved in the denitrosylation of VEGFD at Cys277. A549 cells transfected with WT and Mutant were treated with N91115 and control. Western blot analysis indicated that inhibition of GSNOR suppressed the expression of VEGFD but didn’t repress the mutant (Fig. 5D). These results suggested that GSNOR is involved in the denitrosylation of VEGFD. Subsequently, we explored the function of Trx in the denitrosylation of VEGFD. Trx plays a role in denitrosylation by the synergy of Cys32 and Cys35. We constructed Trx-WT (wild type) and Trx-C32S/C35S mutant (loss of function). The overexpression of Trx-WT could induce the expression of VEGFD, while Trx-C32S/C35S could not reverse the promotion (Fig. 5D). The WB results also suggest that Trx-WT promotes the expression of VEGFD and mutant. Meanwhile, Trx-C32S/C35S could not reverse the function of Trx-WT (Fig. 5E). To explore the potential work of Trx on the other Cys residues of VEGFD, the WB showed that Trx has no effects on other Cys residues (Fig. 5F). These results mean that Trx does not participate in the denitrosylation of VEGFD. Moreover, the Co-IP results showed that VEGFD binds to GSNOR, and the VEGFD mutant weakens its binding to GSNOR (Fig. 5G). However, VEGFD does not combine with Trx. Similar colocalization was observed with either dual fluorescence, whereas the VEGFD mutant reduced colocalization with GSNOR (Fig. 5H). Moreover, the ABE assay showed that GSNOR inhibits S-nitrosylation of VEGFD. Conversely, N91115 reversed the inhibitory effect of GSNOR on VEGFD S-nitrosylation (Fig. 5I). Thence, GSNOR is significant to the denitrosylation of VEGFD at Cys277.

### Inhibition of GSNOR exacerbates LUAD

**Fig. 6:**
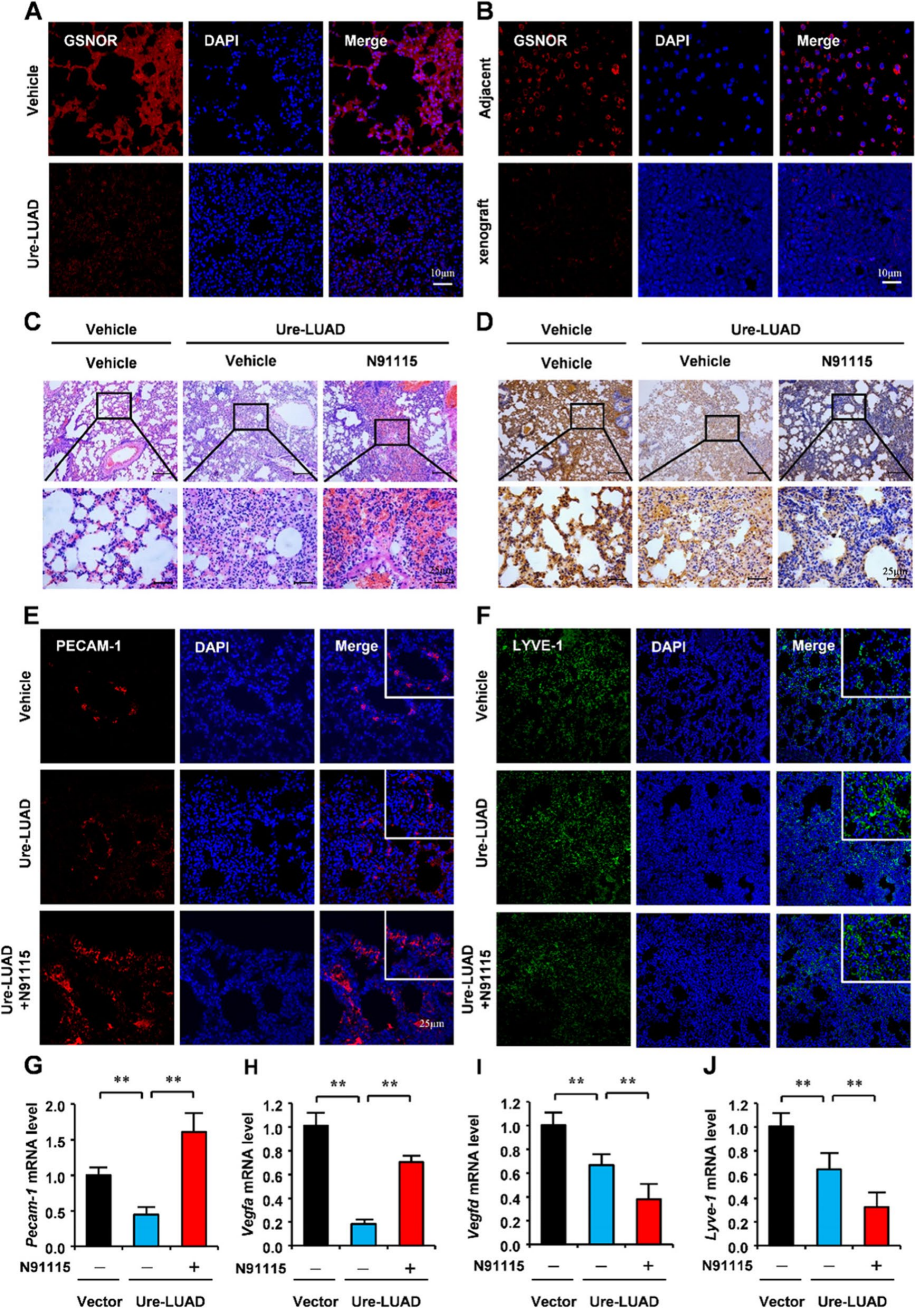
GSNOR-mediated denitrosylation of VEGFD is associated with the development of lung adenocarcinoma. **A**, **B** Immunofluorescence staining for GSNOR in lungs with urethane-induced tumors and in LLC cell xenografts. **C**, **D** H&E and IHC staining for GSNOR in lungs with urethane-induced tumors. **E** Immunofluorescence staining for PECAM-1 in lungs with urethane-induced tumors. **F** Immunofluorescence staining for LYVE-1 in lungs with urethane-induced tumors. **G-J** Quantitative RT-PCR analyses in lungs with urethane-induced tumors. Numerical data were expressed as mean ± SD (each *n* = 5). Data in (**A**), (**B**), (**C**), (**D**), (**E**), and (**F**) are representative of three independent experiments. and data in (**G**), (**H**), (**I**), and (**J**) represent the mean ± SEM of triplicate samples. * *p* < 0.05, **p < 0.01. Student’s t test

To verify whether GSNOR-mediated denitrosylation of VEGFD is involved in LUAD. We analyzed the mRNA and protein level of GSNOR in LUAD by Assistant for Clinical Bioinformatics and UALCAN-CPTAC analysis. The above results showed that the mRNA and protein level of GSNOR in LUAD was significantly decreased (Fig. 2C and D). Western blot results verify the repression of GSNOR in the Ure-LUAD and xenograft model (Fig. 2E F). Meanwhile, GSNOR immunofluorescent staining (green) in the lungs of Ure-LUAD and xenograft also indicates the suppression of VEGFD (Fig. 6A and B). These results further showed the overactivity of S-nitrosylation in LUAD. In the Ure-LUAD model, the C57 mice induced by urethane were treated by N91115 and control. The staining of H&E and Ki67 indicated that N91115 exacerbates LUAD (Figs. 6C, S3A, and B). Meanwhile, Immunohistochemical staining showed that N91115 suppresses VEGFD in the Ure-LUAD model (Fig. 6D). We have pointed out that the inhibition of VEGFD is closely related to the metastasis of LUAD. To clarify the function of inhibition of VEGFD by S-nitrosylation in LUAD metastasis. Immunofluorescence suggested that N91115 facilitates angiogenesis by inhibiting VEGFD (Fig. 6E). However, immunofluorescence also suggested that N91115 did not affect lymphangiogenesis (Fig. 6F). Moreover, the quantitative RT-PCR showed that N91115 could induce angiogenesis-related gene expression while repressing lymphangiogenesis (Fig. 6G H, I, and J). These results indicate that S-nitrosylation of VEGFD exacerbates LUAD via angiogenesis.

Notably, the previous research showed that S-nitrosylation of targets could promote angiogenesis by inducing VEGFA. Similarly, the Western blot results showed that NO promotes VEGFA expression (Fig. S3C). However, we have shown that GSNOR does not affect the expression level of VEGFA (Figs. S2B and S3D). Furthermore, N91115 also had no effects on VEGFA expression (Fig. S3E). Therefore, GSNOR-mediated denitrosylation affects LUAD angiogenesis primarily through VEGFD but not VEGFA. S-nitrosylation may influence VEGFA via other pathways.

### S-nitrosylation of VEGFD is essential for secretion

**Fig. 7:**
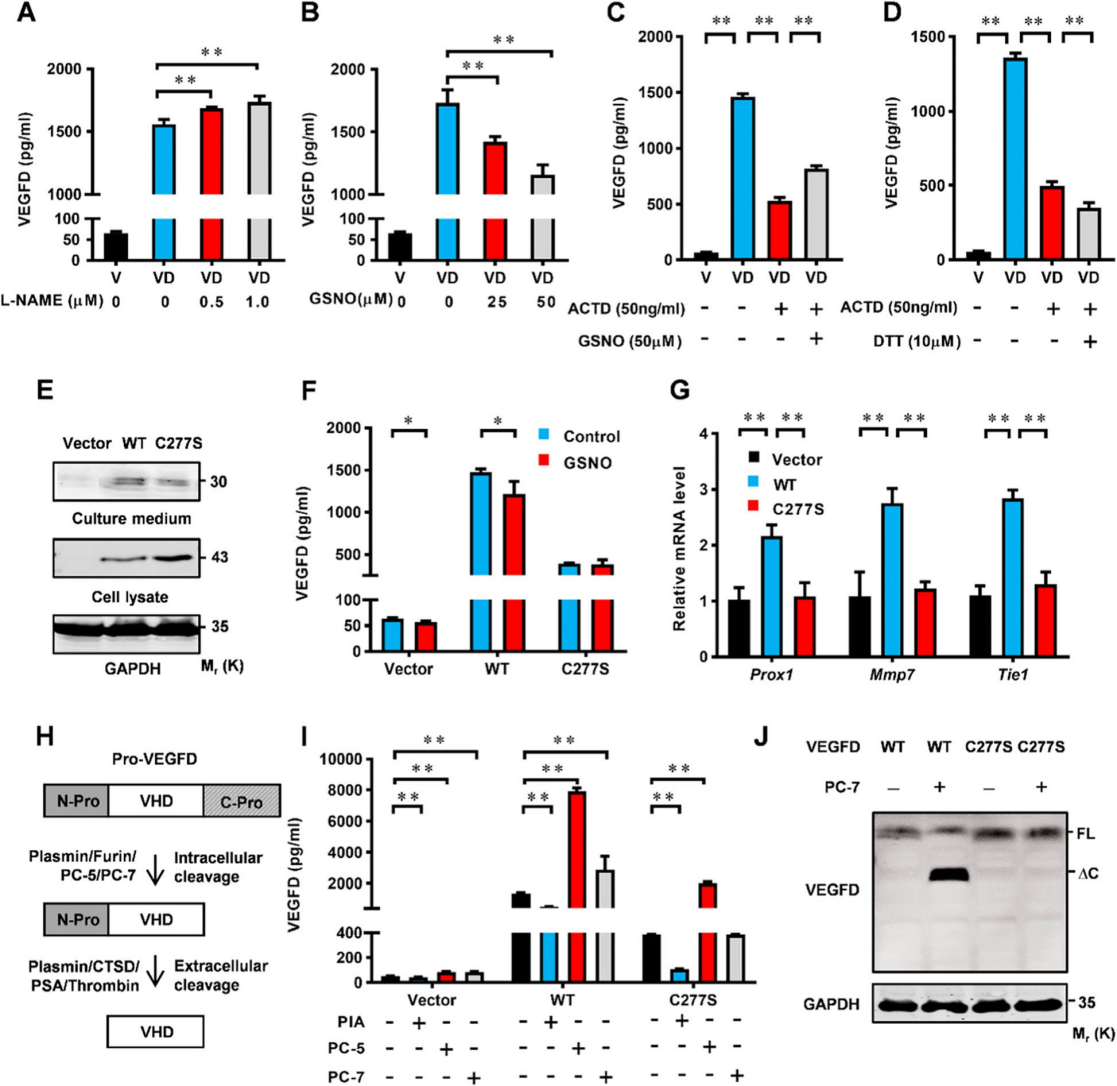
S-nitrosylation of VEGFD affects its secretion. **A**-**B** HEK-293T cells were transfected with vector or VEGFD-expressing construct, cultured for 24 h, and then treated with L-NAME or GSNO for further 24 h. ELISA assays detects secreted VEGFD levels in the culture supernatants. **C**-**D** HEK-293T cells were transfected with vector (V) or HA-VEGFD (VD), pretreated with ACTD for 6 h, and then treated with GSNO at 50µM or DTT at 10µM for further 24 h. ELISA assays detects secreted VEGFD levels in the culture supernatants. (E)Western blot analyses for VEGFD in the culture supernatants and 293T cells transfected with vector, HA-VEGFD (WT) or HA-VEGFD(C277S) mutant and then cultured for 48 h. **F** HEK-293T cells were transfected with WT or C277S mutant, ELISA assays detected secreted VEGFD in the culture supernatants. **G** NCI-H1975 cells were transfected with vector, WT or C277S mutant and then cultured for 24 h, media was collected to treat HUVEC cells for 24 h, and then HUVEC cells were harvested for qPCR. **H** Schematic diagram for the proteolytic cleavages of VEGFD by proteases. **I** ELISA assays for the culture supernatants of 293T cells at 48 h post co-transfection with WT/C277S and PIA/PC-5/PC-7. **J** Western blot analyses in 293T cells at 24 h post co-transfection with WT/C277S and PC-7. Data in (**E**) and (**J**) are representative of three independent experiments. and data in (**A**), (**B**), (**C**), (**D**), (**F**), (**G**), and (**I**) represent the mean ± SEM of triplicate samples. * *p* < 0.05, **p < 0.01. Student’s t test

Furthermore, NO and S-nitrosylation have two faces in cancer [18]. Excessive S-nitrosylation induces tumor development, but S-nitrosylation could also facilitate tumor cell death. Similarly, although excessive S-nitrosylation inhibits VEGFD in LUAD, the function of normal S-nitrosylated VEGFD remain unknown. We treated the NCI-H1975 cells with L-NAME. The cell culture fluid tested by the ELISA kit showed that L-NAME promotes the secreted VEGFD (Fig. 7A). On the contrary, the cell culture fluid tested by the ELISA kit showed that GSNO represses the secreted VEGFD (Fig. 7B). Interestingly, NCI-H1975 cell were treated with GSNO at different doses, the cellular protein and secreted protein levels of VEGFD were inconsistent at the same time and dose (Fig. S4A and B), it indicated that S-nitrosylation may affect secretion of VEGFD. VEGFD is a secreted protein that is cleaved by proteolytic enzymes to form the mature form, which is then secreted outside the cell. However, the previous data indicated that S-nitrosylation suppresses VEGFD at protein level. To avoid the influences of VEGFD transcription levels, cells transfected with VEGFD were treated with Actinomycin D (ACTD) to decrease RNA synthesis. The ELISA results of cell culture fluid indicated that S-nitrosylation could promote the secretion of VEGFD while denitrosylation disrupts the secretion (Fig. 7C and D). Meanwhile, immunofluorescence displayed that the VEGFD^C277S^ mutant was significantly increased in the cytoplasm (Fig. S4C). Proteins in the culture medium were concentrated by TCA precipitation and Western blot results showed the reduction of secretion in the VEGFD^C277S^ mutant (Fig. 7E). We used an ELISA kit to detect secreted VEGFD, the ELISA results of cell culture fluid indicated that the VEGFD^C277S^ mutant decreased secretion (Fig. 7F). The secretion of VEGFD is significant to angiogenesis and lymphangiogenesis, PROX1, TIE1, and MMP7 are target genes of VEGFD in angiogenesis and lymphangiogenesis. The quantitative RT-PCR results showed that the VEGFD^C277S^ mutant deactivates the target genes (Fig. 7E). To verify the effect of the VEGFD^C277S^ mutant on its function, we conducted a tube formation assay by HUVEC cells in vitro. HUVEC cells were transfected with Vector, WT, and C277S mutant, respectively, and tube formation assays showed that the VEGFD^C277S^ mutant was dysfunctional (Fig. S4D, E, and F). These results suggest that normal S-nitrosylation is essential for the secretion of VEGFD.

As the mechanism of S-nitrosylation affects secretion, there are multiple proteolytic enzymes, including CTSD, KLK3, Plasmin, Furin, PC5, and PC7 [42–44]. The expression of these proteolytic enzymes is related to the maturation and secretion of VEGFD. To explore the functions of S-nitrosylation on proteolytic enzymes, we treated BASE-2B cells with L-NAME. The quantitative RT-PCR results show that proteolytic enzymes of VEGFD are activated (Fig. S3A-F). On the contrary, BASE-2B cells were stimulated with GSNO, and the expression of these enzymes was significantly decreased (Fig. S3G-L). To further validate our results, HEK-293T cells transfected with PAI/PC-5/PC-7 were treated with GSNO (10µM), and the ELISA assay showed that proteolytic-activated VEGFD is inhibited by GSNO (Fig. S3M-O). These results indicated that NO affects the expression of VEGFD-related proteolytic enzymes. Proteolytic cleavages of VEGFD require two concerted proteolytic cleavages (Fig. 7H) The intracellular cleavage occurs between the central VEGF homology domain (VHD) and the C-terminal propeptide. protein convertases constitutively cleave VEGF-D before secretion. The extracellular cleavage occurs between the N-terminal propeptide and the VHD and can be mediated by different proteases [45]. We speculate that the VEGFD^C277S^ mutant cannot be cleaved by proteolysis. There are loads of proteases in the proteolysis of VEGFD C-terminal propeptide [46]. To further explore the mechanism of S-nitrosylation regulating secretion of VEGFD, we co-transfected HEK-293T cells with VEGFD/VEGFD^C277S^ and PAI/PC-5/PC-7, the ELISA results suggest that the VEGFD^C277S^ mutant couldn’t be influenced by PC-7 not Plasmin/PC-5 (Fig. 7I). Western blot results showed the same results (Fig. 7J), indicating that the VEGFD^C277S^ mutant disrupts PC-7-dependent proteolysis. Plasmin and PC-5 can hydrolyze carboxyl-terminal and N-terminal amino acids and of VEGFD. However, PC-7 is a proteolytic enzyme that only hydrolyzes carboxyl-terminal amino acids of VEGFD [46]. These results suggest that S-nitrosylation of VEGFD is responsible for PC-7-dependent proteolysis.

### VEGFD requires S-nitrosylation to repress LUAD via angiogenesis

**Fig. 8:**
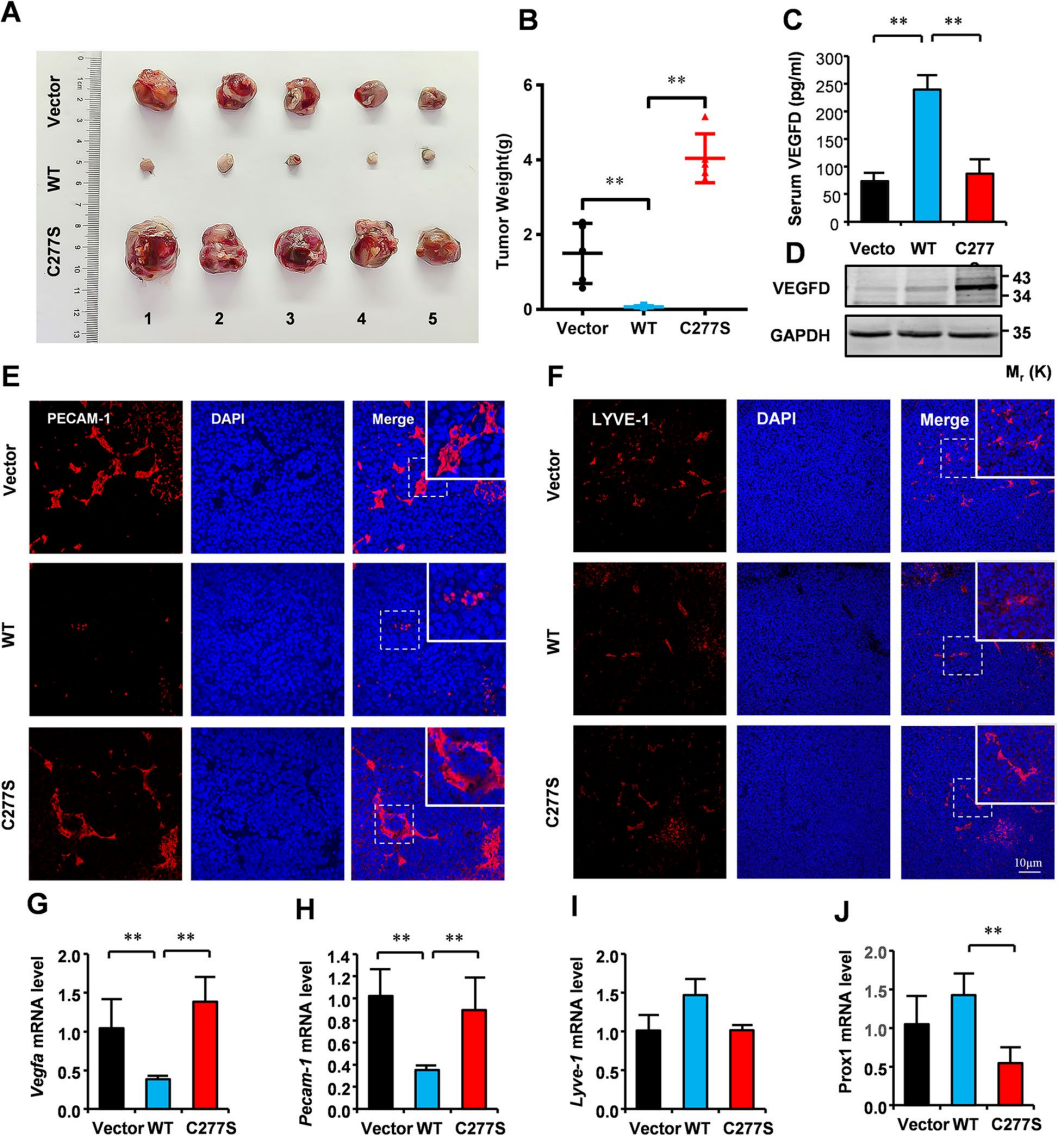
Denitrosylation of VEGFD disrupts its tumor suppressor functions. On day 24 post-inoculation, C57/BL6 mice bearing the Vector-, WT- or C277S-expressing LLC cell xenografts were used for the following assays. **A**-**B** Xenografts were harvested for imaging and weighing. **C** ELISA assay of VEGFD levels in the sera of C57/BL6 mice. **D** Western blot analyses in xenografts. **E**-**F** Immunofluorescence staining for PECAM-1 and LYVE-1 in the paraffin-embedded xenografts. (Q-J) Quantitative RT-PCR analyses in xenografts. Numerical data were expressed as mean ± SD (each *n* = 5). Data in (**D**) are representative of three independent experiments. and data in (**B**), (**C**), (**G**), (**H**), (**I**), and (**J**) represent the mean ± SEM of 5 samples. * *p* < 0.05, **p < 0.01. Student’s t test

We have revealed the mechanism by which S-nitrosylation regulates VEGFD, which is associated with LUAD. To further verify the role of VEGFD S-nitrosylation in LUAD, we generated LLC cells stably expressing Vector, WT, or C277S mutant, which were injected into the axillary of C57 mice to generate LLC cell xenografts. After 24 days post-inoculation, the volume and weight of xenografts expressing WT were decreased by 90% compared with those expressing Vector, whereas the volume and weight of xenografts expressing C277S mutant were reverse the reduction of those expressing WT (Fig. 8A and B). Moreover, analyses of cell apoptosis in xenografts indicated overexpression of WT increased the TUNEL-positive cells, as compared with overexpression of Vector, whereas overexpression of C77S decreased the TUNEL-positive cells compared with overexpression of WT (Fig. S6A and B). These suggest that VEGFD inhibits the tumorigenesis of LUAD, and the C277S mutant can reverse the inhibition of LUAD. The ELISA results of xenografts serum indicated that xenografts expressing WT increased secretion of VEGFD while the VEGFD^C277S^ mutant decreased secretion (Fig. 8C). Meanwhile, the WB results showed the xenografts expressing C277S mutant increased VEGFD compared with those expressing WT (Fig. 8D). Those were consistent with the results in vitro. The VEGFs generally affect tumorigenesis and metastasis through angiogenesis and lymphangiogenesis. The immunofluorescence of xenografts sections suggests that angiogenesis in xenografts expressing WT is significantly reduced (Fig. 8E). And there was no obvious change in the lymphatic vessels of xenografts expressing Vector, WT, and C277S mutant (Fig. 8F). The qRT-PCR results showed that the expression of angiogenesis-related genes (Vegfa and CD31) in xenografts expressing WT was significantly reduced than those expressing Vector and C277S mutant (Fig. 8G H). The lymphangiogenesis-related genes (Lyve-1 and Prox-1) were no significant change in xenografts expressing WT compared with those expressing Vector (Fig. 8I J). To prove a pro-migratory effect in relation to angiogenesis, a wound healing assay was performed to examine the effects of secreted proteins from NCI-H1975 cells on HUVEC cell migration. We found that NCI-H1975 cells expressing VEGFD reduced HUVEC cells migration, but NCI-H1975 cells expressing C277S mutant enhanced migration (Fig. S6C and D). Similarly, the transwell migration assay showed the same results (Fig. S6E and F). These results suggest that VEGFD may inhibit LUAD by disrupting angiogenesis.

VEGFD induces angiogenesis through binding with VEGFR2. But the effects of VEGFD in suppressing LUAD by disrupting angiogenesis are inconsistent with the classic theory. Niki et al. pointed out that the ratios of VEGF-D: VEGF-A, VEGF-D: VEGF-B, or VEGF-D: VEGF-C were significantly lower in the node-positive group of LUAD [47]. VEGFA, VEGFC, and VEGFD can bind with VEGFR2 to induce angiogenesis. Thence, we speculate the balance between VEGFD and VEGFA/VEGFC could influence the angiogenesis of LUAD. To verify our speculation, we investigated the expression of VEGFA and VEGFC in LUAD by Assistant for Clinical Bioinformatics and UALCAN-TCGA analysis. The mRNA level of both VEGFA and VEGFC was reduced in LUAD (Fig. S7A and B). However, the protein level of VEGFA is increased in LUAD (Fig. S7C), and the protein level of VEGFC has no change (Fig. S7D). The correlation analysis by GEPIA shows that VEGFD and VEGFA is a negative correlation in LUAD while VEGFD and VEGFC do not correlate (Fig. S7E and F). We guess whether the balance of VEGFD and VEGFA is significant to the tumorigenesis and metastasis of LUAD. VEGFD and VEGFA both binds with VEGFR2 to induce angiogenesis. Li et al. revealed that S-nitrosylation is a vital regulator of radiation-induced HIF-1α activation, which could boost VEGFA expression. We show the suppression of VEGFD by S-nitrosylation. All in all, we speculate that S-nitrosylation could suppress VEGFD and induce VEGFA, which disrupts the balance between VEGFA and VEGFD. VEGFA is the main incentive of angiogenesis. When VEGFD is inhibited, more VEGFA bind with VEGFR2 exaggerates angiogenesis. On the contrary, overexpression of VEGFD limits VEGFA binds to VEGFR2. To verify our guess, Co-immunoprecipitation experiments show that when VEGFD is overexpressed, the binding of VEGFR2 to VEGFD increases, but the binding to VEGFA decreases (Fig. S7G). When VEGFD is mutated at Cys277, the combination of VEGFR2 to VEGFD decreases, and the combination to VEGFA increases (Fig. S7H and I). To evaluate the effects of mutant solubility and biochemical properties, we predicted the solubility of mutant via Protein-Sol webserver [48]. The results showed that VEGFD mutants do not affect their solubility (Fig. S7J). Based on the above results, we propose a theory that VEGFD can inhibit angiogenesis by disrupting the balance between VEGFD and VEGFA, which reduces metastasis. Moreover, Schmeisser et al. found that VEGFD mediates monocyte/macrophage apoptosis [49]. We speculate that VEGED may have an anti-tumor function by inducing apoptosis. In summary, We provide two theories to explain the function of VEGFD on LUAD. VEGFD inhibits angiogenesis of LUAD by competing with VEGFA to bind to VEGFR2. Moreover, VEGFD also induces tumor apoptosis. We speculate that the dual action of VEGFD in lung cancer represses tumorigenesis of LUAD, which is our next research scope.

## Discussion

The tumorigenesis and metastasis are associated with angiogenesis and lymphangiogenesis. VEGFs play significant roles in angiogenesis and lymphangiogenesis [3]. Among them, VEGFA is the main angiogenic factor. VEGFC is responsible for lymphangiogenesis. VEGFD could partake in angiogenesis and lymphangiogenesis. However, VEGFD is dispensable for lymphangiogenesis. In pathological conditions, VEGFD was always thought to promote tumors by lymphangiogenesis [7]. However, several studies pointed out the suppression of VEGFD in tumors [26, 47]. Here, we found that the inhibition of VEGFD by S-nitrosylation was significantly higher than that of NO-regulated *VEGFD* mRNA; for the mechanism of NO inhibiting the transcription of VEGFD, it is the consensus that NO inhibits C-Jun [50], and C-Jun promotes the transcription of VEGFD [51], which can be inferred NO interferes with the transcription of VEGFD by inhibiting C-Jun. Here we found that the effect of S-nitrosylation on VEGFD was stronger than that of transcription, so we mainly explored the effect of S-nitrosylation on VEGFD.

The carcinogenic effects of S-nitrosylation are well known. LUAD is a subtype of NSCLC. S-nitrosylation could promote LUAD by repressing VEGFD and facilitating VEGFA. There is no evident correlation between VEGFA expression and LUAD. Instead, the low expression of VEGFD is significantly related to the LUAD. Our findings suggest that VEGFD could be a new biomarker of LUAD. Meanwhile, VEGFA and VEGFD bind to VEGFR2 to activate angiogenesis. Inhibition of VEGF/VEGFR2 by monoclonal antibodies is always the hotpot of anti-tumor therapy. Bevacizumab, a monoclonal antibody targeting VEGF, could suppress the functions of VEGF. It could decrease the angiogenesis and metastasis of tumors. Food and Drug Administration (FDA) Approved Bevacizumab for multiple tumors, including NSCLC, Colorectal cancer, ovarian cancer, and so on [52]. In the NSCLC, Bevacizumab treats advanced NSCLC [53]. However, there are various side effects of it. Pulmonary hemorrhage is frequent in NSCLC. Our research may provide a new strategy for LUAD therapy by denitrosylation mediated the balance of VEGFD and VEGFA. Denitrosylation could inhibit angiogenesis by reversing the suppression of VEGFD and repressing VEGFA. Moreover, VEGFD could avoid pulmonary hemorrhage because it has a pro-angiogenesis effect.

Meanwhile, GSNOR mediates the denitrosylation of VEGFD. Inhibiting GSNOR by N91115 exaggerates LUAD through repressing VEGFD. GSNOR has a significant role in the balance of S-nitrosylation and denitrosylation. The suppression of GSNOR in the LUAD disrupts the balance and promotes S-nitrosylation. The S-nitrosylation of VEGFD induces the development of LUAD. GSNOR has tumor suppressor roles. Defects and deficiencies in the GSNOR have been associated with the development and malignancy of liver and breast cancers [54, 55]. We have confirmed the functions of GSNOR on the angiogenesis of LUAD. Inhibition of GSNOR promotes LUAD. Instead, activation of GSNOR promotes denitrosylation which could facilitate VEGFD and inhibit VEGFA. It may be a potential target in the treatment of LUAD.

## Conclusion

**Fig. 9:**
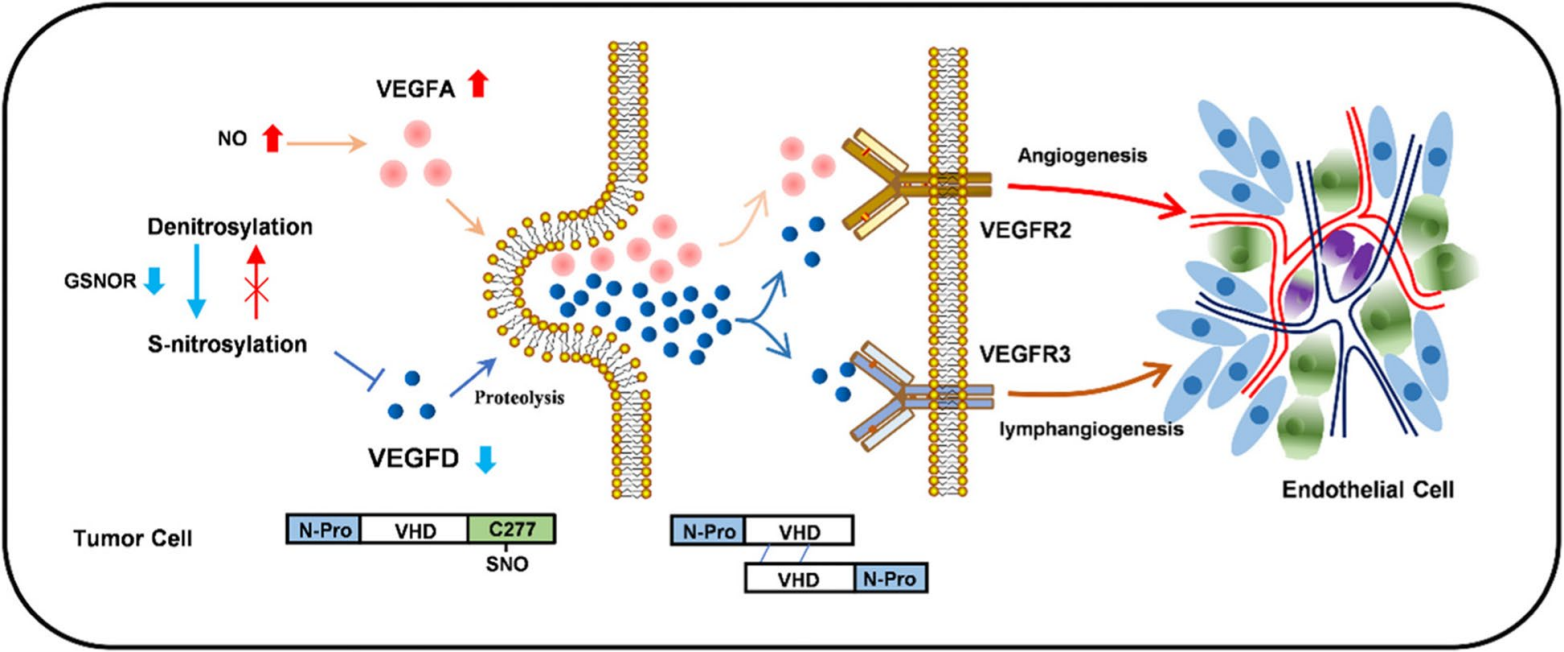
An integrated working model for S-nitrosylation-negated VEGFD in lung adenocarcinoma. Excessive NO and low expression of GSNOR in lung adenocarcinoma exacerbate S-nitrosylation of VEGFD at Cys277, suppressing its expression by affecting stability, while S-nitrosylation of VEGFD also affects its secretion, meanwhile NO promotes VEGFA, VEGFA and VEGFD compete for binding to VEGFR2 to promote angiogenesis

In summary, we found that excessive NO and inhibition of GSNOR in LUAD promote S-nitrosylation of VEGFD, and S-nitrosylation of VEGFD at Cys277 inhibits VEGFD protein level in LUAD; inhibition of VEGFD is associated with the tumorigenesis and metastasis of LUAD. However, VEGFD also requires normal S-nitrosylation to repress LUAD angiogenesis, as S-nitrosylation is indispensable for the secretion of VEGFD (Fig. 9). Furthermore, VEGFD inhibits LUAD angiogenesis by competing with VEGFA to bind to VEGFR2 in LUAD, and it also induces tumor apoptosis, which is our next research scope.

## Supplementary Information

**Additional file 1.**

## Data Availability

All publicly available data can be acquired from the corresponding web servers described in the Materials and methods. The data used and/or analyzed in the present study are available from the corresponding author on reasonable request.

## Abbreviations

VEGF: Vascular endothelial growth factor
LUAD: Lung adenocarcinoma
GSNOR: S-nitrosoglutathione reductase
Trx/TrxR: Thioredoxin/thioredoxin reductase
ACTD: Actinomycin D
SNP: Sodium nitroprusside
NSCLC: Non-small cell lung cancer
PLGF: Placental growth factor
NO: Nitric oxide
PTM: Post-translational modification
TCGA: The Cancer Genome Atlas
CPTAC: Clinical Proteomic Tumor Analysis Consortium
Ure: Urethane
LPS: Lipopolysaccharide
CTSD: Cathepsin D
PAI: Plasminogen activator inhibitor
PC5: Proprotein convertase subtilisin/kexin type 5
PC7: Proprotein convertase subtilisin/kexin type 7
