# Supplementary material for: Suppression of VEGFD expression by S-nitrosylation promotes the development of lung adenocarcinoma
Source: J Exp Clin Cancer Res. 2022 Aug 8;41:239. doi: 10.1186/s13046-022-02453-8 (PMC9358865; doi:10.1186/s13046-022-02453-8)
Supplement: Supplementary file 1 — Additional file 1. [file 13046_2022_2453_MOESM1_ESM.docx]

Supplementary Figures

**Figure S1**


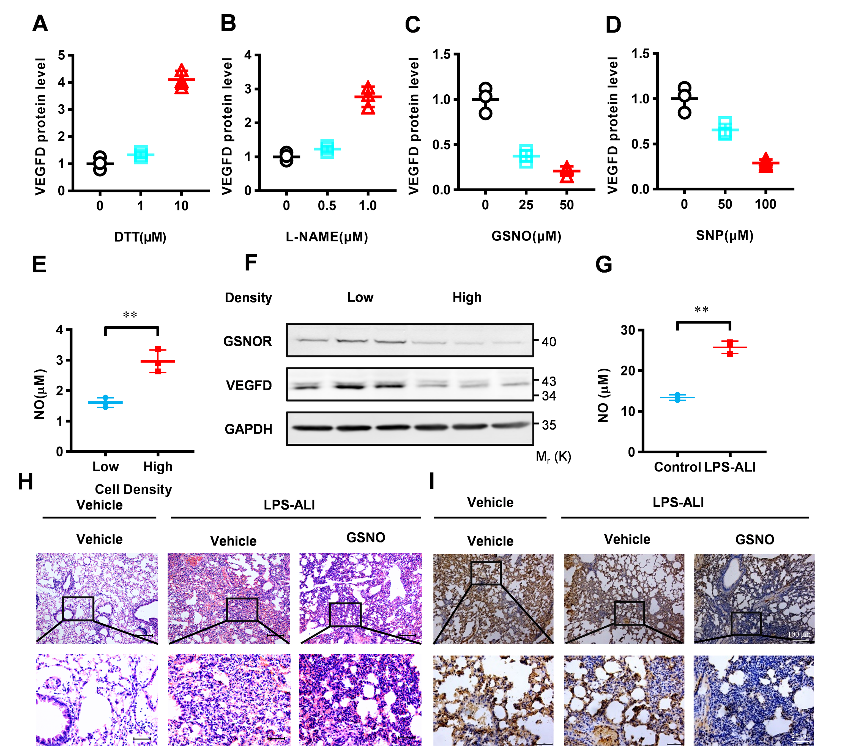


**Figure S1. Increased NO suppresses VEGFD in vivo and in vitro.** (A-D) Quantification of WB analysis for **Fig 2G-J** through Image J. Numerical data were expressed as mean ± SD (each n = 3). *p<0.05, **p<0.01. Tukey-Kramer multiple comparison test. (E) NO assay of BASE-2B cells in low and high cell density. (F) Western blot analysis of VEGFD and GSNOR in low and high cell density. (G) NO assay of LPS-induced acute lung injury (ALI). (H) The H&E staining of LPS-induced acute lung injury. (I) The IHC for VEGFD in LPS–induced acute lung injury. Data in (F), (H), and (I) are representative of three independent experiments, and data in (E) and (G) represent the mean ± SEM of triplicate samples. *P < 0.05, **P < 0.01, Student’s t test.

**Figure S2**


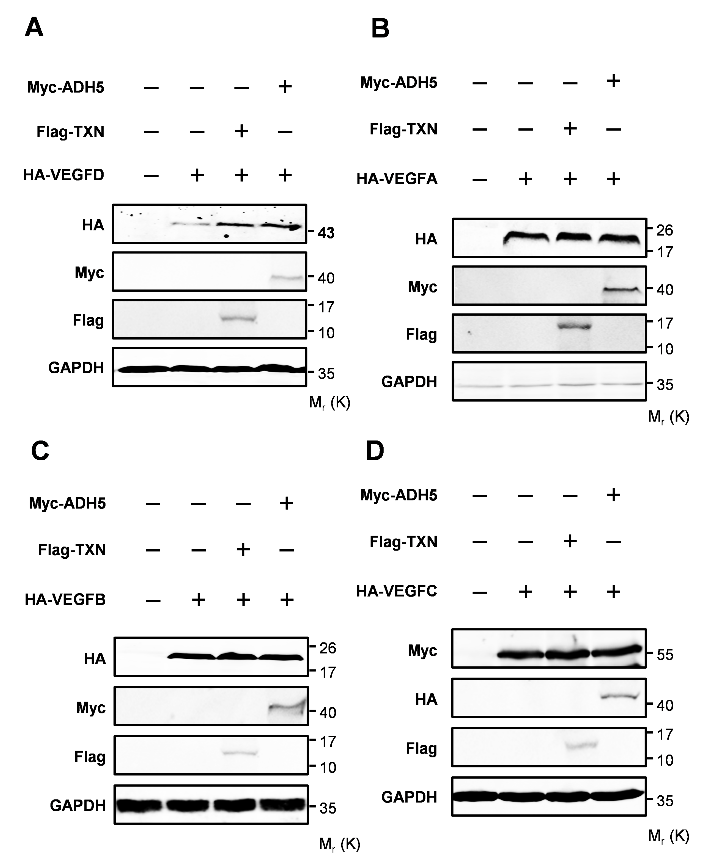


**Figure S2. The impacts of S-nitrosylation on the VEGF family.** (A-D) HEK-293T cells were transfected with HA-VEGFD/VEGFA/VEGFC/VEGFD in combination with Flag-TXN or Myc-ADH5, cell extract was harvested 48h after transient transfection and processed for Western Blot analysis. Data in (A), (B), (C), and (D) are representative of three independent experiments.

**Figure S3**


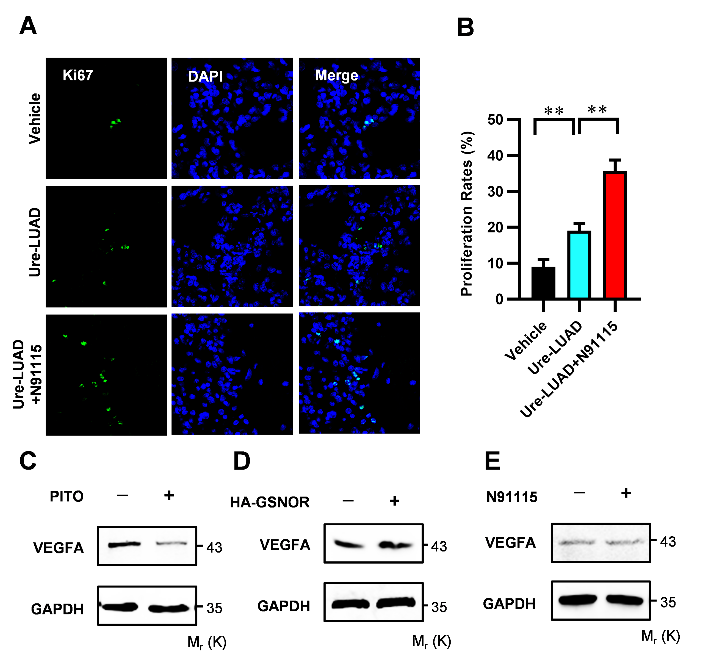


**Figure S3. N91115 exacerbates lung adenocarcinoma but does not affect VEGFA expression.** (A-B) Lungs of urethane-induced lung tumors were used for sectioning, staining of Ki67, and their quantification. Numerical data were expressed as mean ± SD (each n = 5). (C) NCI-H1975 cells were treated with PITO (5mM) for 24h, Western Blot analysis VEGFA expression. (D) NCI-H1975 cells were transfected with Myc-ADH5, and VEGFA expression was analyzed by Western blot 48 hours later. (E) NCI-H1975 cells were treated with N91115 (5mM) for 24h, Western Blot analysis VEGFA expression. Data in (C), (D), and (E) are representative of three independent experiments, and data in (B) represent the mean ± SEM of triplicate samples. *P < 0.05, **P < 0.01, Student’s t test.

**Figure S4**


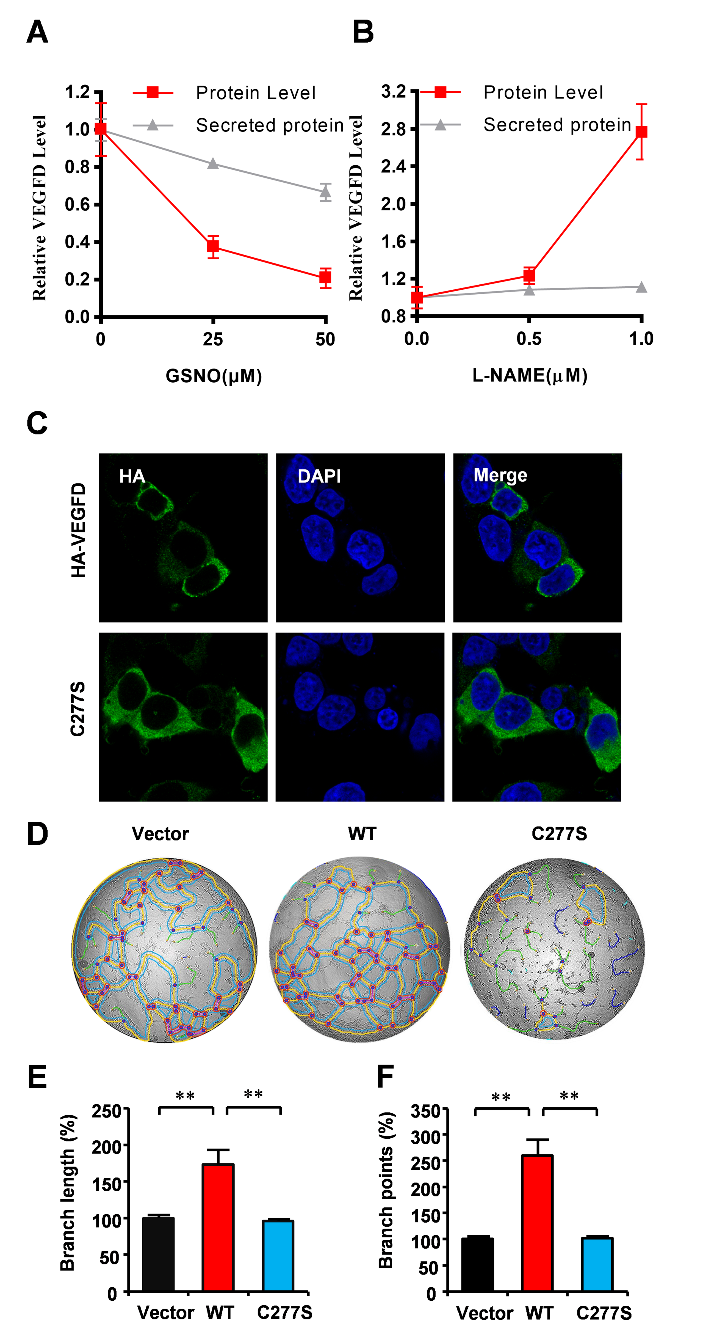


**Figure S4. S-nitrosylation of VEGFD at Cys277 affects its secretion and tube formation function.** (A-B) NCI-H1975 cells treated with GSNO or L-NAME. quantification of WB analysis for VEGFD, and ELISA analyses for VEGFD in culture fluids. Numerical data were expressed as mean ± SD (each n = 3). *P < 0.05, **P < 0.01, Student’s t test. (C) IF staining in NCI-1975 cells at 48 h post-transfection with HA-VEGFD and HA-VEGFD(C277S) mutant. (D) tube formation assay by HUVEC transfected with Vector, WT, and C277S mutant. (E-F) The branch length and points statistics by Image J software.

**Figure S5**


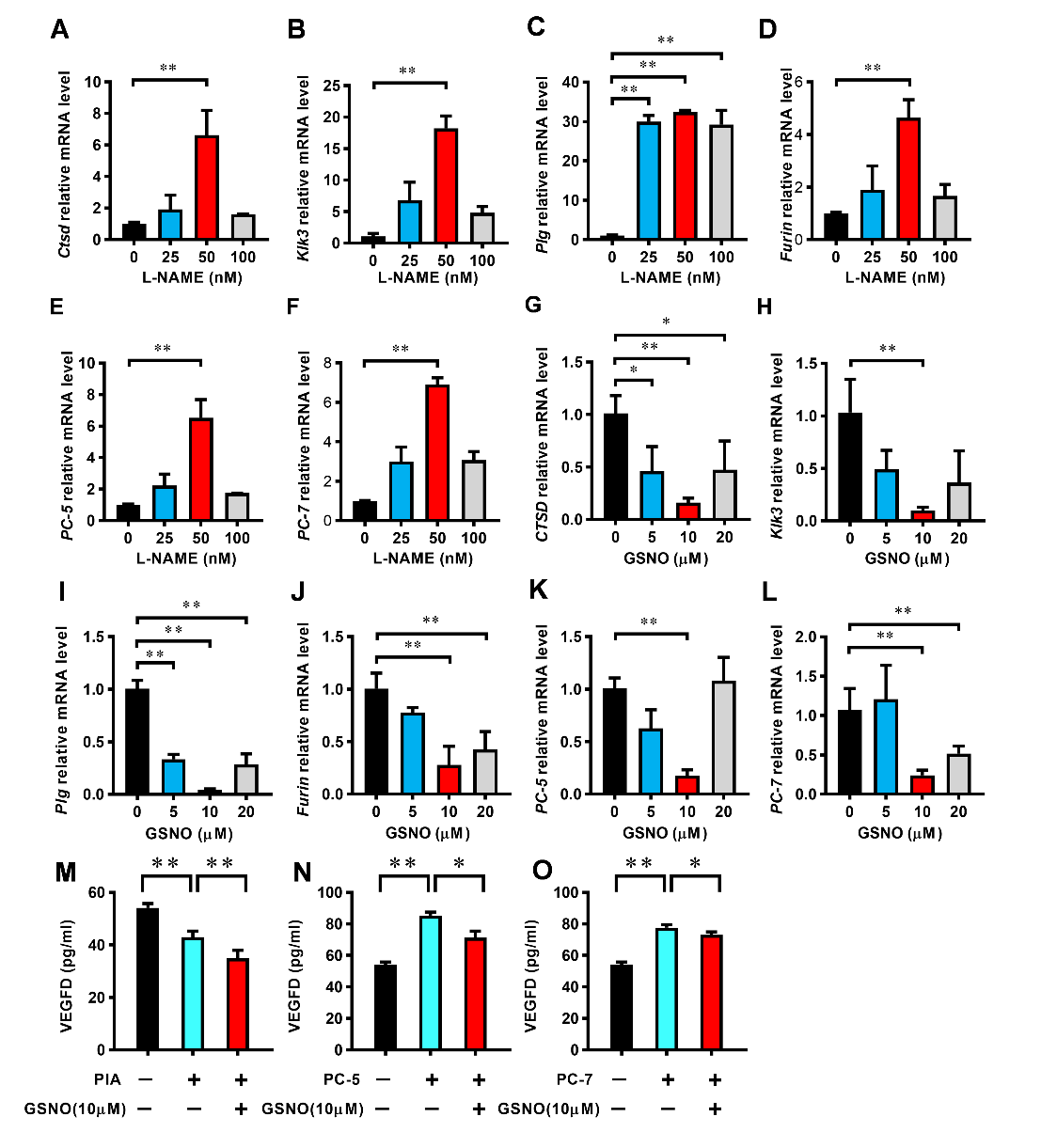


**Figure S5. S-nitrosylation suppresses VEGFD-related protease expression.** (A-F) The NCI-H1975 cells treated with L-NAME for 24h were used for isolation of total RNA for qPCR of VEGFD-related proteases. (G-L) The NCI-H1975 cells treated with GSNO for 24h were used for isolation of total RNA for qPCR of VEGFD-related proteases. (M-O) HEK-293T cells were transfected with PIA/PC-5/PC-7 for 24h and then treated with GSNO at 10μM for 24h. ELISA assays detect secreted VEGFD levels in the culture supernatants. Numerical data were expressed as mean ± SD (each n = **3**). *p<0.05, **p<0.01. Tukey-Kramer multiple comparison test.

**Figure S6**


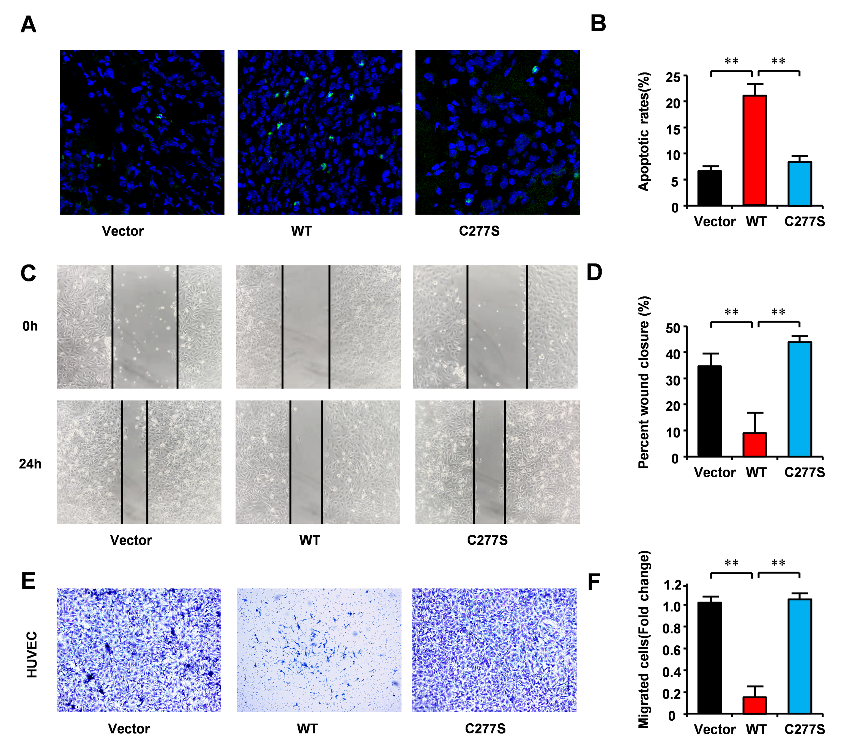


**Figure S6. The VEGFD^C277S^ mutant affects** **its repression on LUAD.** (A-B) Xenografts were used for sectioning, staining of TUNEL, and their quantification. Numerical data were expressed as mean ± SD (each n = 5). (C-D) The NCI-H1975 cells transfected with Vector, WT, and C277S mutant were seeded in the upper chamber of 0.4 μm, and the HUVEC cells suspension were seeded on a 24-well plate, cells were co-cultured for 24h. Wound healing assay of HUVEC cells. (E-F) Transwell migration assay. HUVEC cells were seeded in the upper chamber of 8 μm, and then the NCI-H1975 cells transfected with Vector, WT, and C277S mutant were seeded on a 24-well plate, After incubation for 24h. data in (B), (D), and (F) represent the mean ± SEM of triplicate samples. *P < 0.05, **P < 0.01, Student’s t test.

**Figure S7**


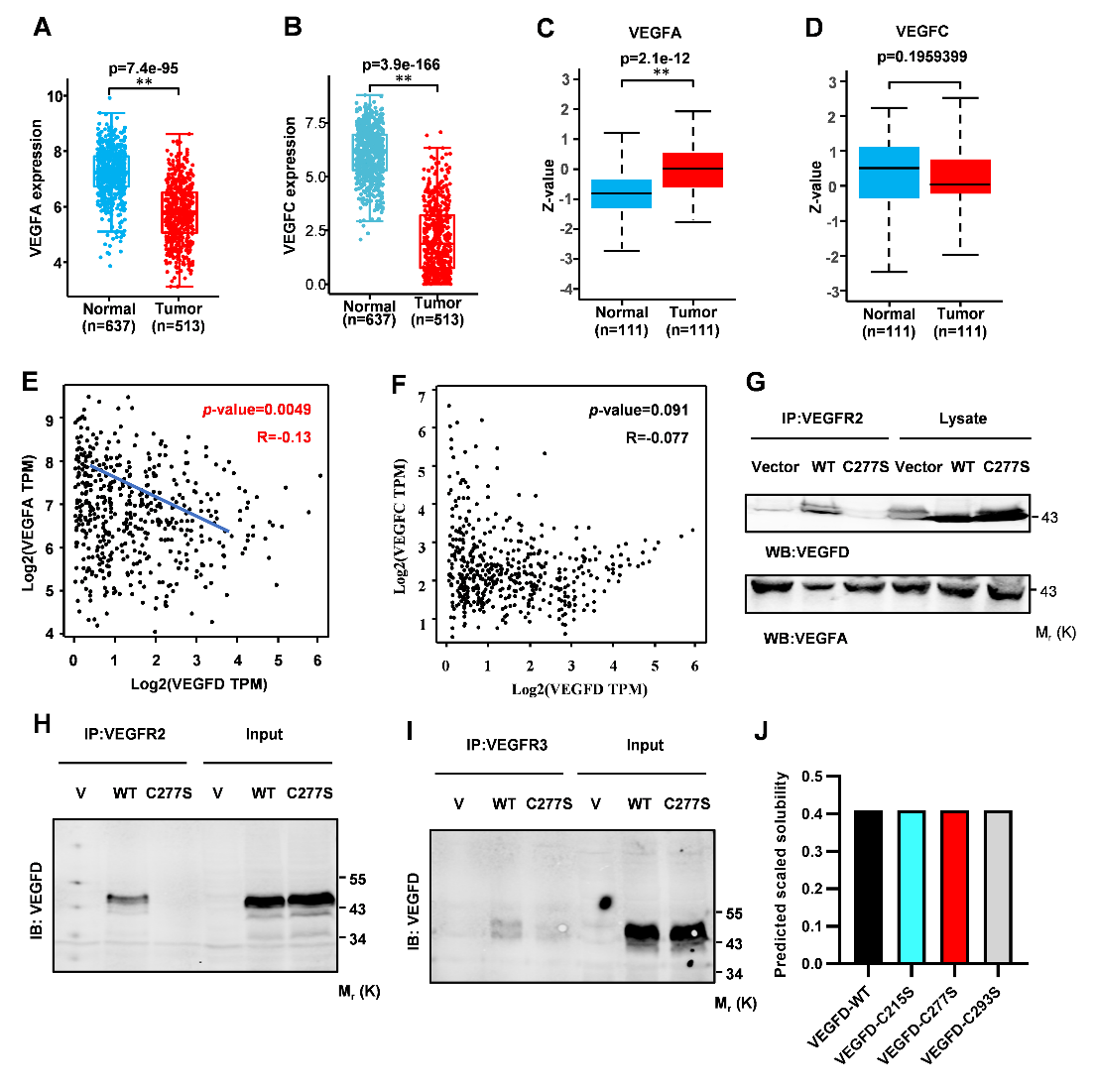


**Figure S7. VEGFD inhibits tumorigenesis by competing with VEGFA to bind to VEGFR2.** (A, B) The mRNA level of VEGFA and VEGFC in lung adenocarcinoma. (C, D) The Protein level of VEGFA and VEGFC in lung adenocarcinoma. (E) The correlation analysis of VEGFA and VEGFD in lung adenocarcinoma. (F) The correlation analysis of VEGFC and VEGFD in lung adenocarcinoma. (G) Co-immunoprecipitation analyses in HEK-293T cells after transfection with HA-VEGFD (WT) and HA-VEGFD^C277S^ mutant (C277S). (H-I) Co-immunoprecipitation analyses by using VEGFR2 or VEGFR3 antibody in NCI-H1975 cells at 24 h post-transfection with WT or C277S mutant. (J) Predicted solubility of VEGFD mutant via Protein-Sol webserver.
